# Supplementary material for: When is a handaxe a planned-axe? exploring morphological variability in the Acheulean
Source: PLoS One. 2024 Jul 16;19(7):e0307081. doi: 10.1371/journal.pone.0307081 (PMC11251633; doi:10.1371/journal.pone.0307081)
Supplement: S1 File — S1 Table. Supplementary Data from the EFA PCA, additional methodological data, and descriptions of the sites included in the analysis. (PDF) [file pone.0307081.s001.pdf]

**When is a handaxe a planned-axe? Exploring morphological variability in the Acheulean**

**Supplementary Information**

**Table of Contents**

*Supplementary Information 1: Data and Tables* ..........1
1. Data .......... 1
2. Tables .......... 1
*Supplementary Information 2: Methodology* ..........3
1. Photographic procedure .......... 3
2. Image Processing .......... 4
3. Measurement Correction .......... 5
*Supplementary Information 3: Site Descriptions*..........14
1. African Sites..........14
2. Levantine Sites..........22
3. European Sites..........27
*Supplementary References*..........44

## Supplementary Information 1: Data and Tables

### 1. Data

Coordinate and measurement data from this investigation can be found in files SIa and SIb.

### 2. Tables

| PC | Eigenvalue | % Variance | Cumulative % |
|---|---|---|---|
| 1 | 0.01267 | 66.430 | 66.43 |
| 2 | 0.002582 | 13.538 | 79.97 |
| 3 | 0.001387 | 7.274 | 87.24 |
| 4 | 0.000432 | 2.265 | 89.51 |
| 5 | 0.000371 | 1.947 | 91.45 |
| 6 | 0.00027 | 1.417 | 92.87 |
| 7 | 0.00021 | 1.102 | 93.97 |
| 8 | 0.000181 | 0.948 | 94.92 |
| 9 | 0.000136 | 0.712 | 95.63 |
| 10 | 0.000119 | 0.623 | 96.26 |
| 11 | 8.26E-05 | 0.433 | 96.69 |
| 12 | 7.16E-05 | 0.376 | 97.07 |
| 13 | 6.66E-05 | 0.349 | 97.41 |

| | | | |
|---|---|---|---|
| 14 | 6.22E-05 | 0.326 | 97.74 |
| 15 | 4.85E-05 | 0.254 | 97.99 |
| 16 | 4.16E-05 | 0.218 | 98.21 |
| 17 | 3.31E-05 | 0.174 | 98.39 |
| 18 | 3.03E-05 | 0.159 | 98.55 |
| 19 | 2.32E-05 | 0.122 | 98.67 |
| 20 | 2.24E-05 | 0.117 | 98.78 |
| 21 | 2.00E-05 | 0.105 | 98.89 |
| 22 | 1.94E-05 | 0.102 | 98.99 |
| 23 | 1.69E-05 | 0.088 | 99.08 |

Table S1. Eigenvalues and percentage of shape variance explained by the first 23 principal components of the EFA PCA.

**Supplementary Information 2: Methodology**

**1. Photographic procedure**

Photographs of handaxes collected by JC for the present study were taken with a Sony DSC-WX350 digital camera with no lens attachment (4896x3672 resolution, 8.56mm focal length, and f/4.5 aperture). Following Iovita and McPherron (2011) and Iovita et al. (2017), handaxes were oriented with their tip to the right of the image, and the most convex side facing up. In some cases it was difficult to distinguish between a tip and a base for specimens which had been worked around the entire circumference, possibly giving rise to a source of random error (Iovita and McPherron, 2011), with final tip designation decided in such cases decided by most prominent deviation from the overall contour and/or smallest cutting angle. A similar problem was encountered when attempting to establish the most convex face of the handaxe, as many appeared to have equal convexity, in which the resting side was selected to be that which allowed for the most horizontal orientation of the cutting edge.

All handaxes were placed in a 40x40x40cm photography box, with a white background, atop a 7cm-high perspex platform. This platform was covered in a clouded sticker to allow enough light through to limit shadow formation, but while preventing excess reflection of light in the photographs. Inside the box, the elongated LED light sources were fixed above and below the handaxe in the plan-view plane, with a diffuser attached underneath to soften the light as it fell on the handaxe.

Photographs were taken from above the handaxe (plan view) and from the side of the handaxe (side view). In each case, the camera was kept 35cm away from the nearest surface of the handaxe, and was set on an optical zoom of 2.0x. This was achieved for plan photographs through the use of a photographic arm kept 42cm above the base of the photography box, clamped to the table upon which the photography box sat and positioned with a ruler. The 10cm photographic scale was affixed onto the platform using white-tack, leaving it below the handaxe in the horizontal plane. The photograph was taken through a circular hole in the top of the photography box with the side shut. For the side-view images, the camera was rested on a perspex stand to create a distance of 7.5cm from the surface to the lens. This was done to place the camera approximately level with the intersection of the two faces of the handaxe. The top of the box was kept closed and the front kept open, with the photographic scale stuck with white-tack onto the front of the platform.

While drawings for the Kapthurin Formation could be downloaded through screen capture software, directly from Leakey et al.'s (1969) paper, digital copies of the monographs did not exist for Olduvai Bed II (Leakey, 1971) and Kalambo Falls (Clark, 2001). As access to a flatbed scanner was not available during data collection for this purpose, the handaxe, chopper, and discoid drawings illustrated in these sources were converted into images using a similar photographic setup. In this case, the books were open to the desired pages and placed directly into the photography box, with the camera was placed 42 cm above the base of the box. The side of the photography box was left open to allow for a manual straightening of the pages during image-taking, in order to minimise the effect of distortion

that may result from this less-than-ideal setup. Images were subsequently split into plan-view and side-view drawings, with the former focused on the more convex side of the artefact facing up, where this was possible.

## 2. Image Processing

### 2.1. Image Rotation

Following Costa (2010), plan-view handaxe images were rotated according to Callow's (1976) method, in which handaxes are oriented about their length axis so that the maximum orthogonal distance from this axis to the edge of the handaxe is equal for each lateral. This method has been shown to be successful at maximising bilateral symmetry in a way that is similar to algorithms designed for this purpose (McPherron and Dibble, 1999). For this process, plan-view images were opened in GIMP-2.10 with a grid superimposed upon the handaxe. The tip of the handaxe was then located and the image rotated about this point to achieve the required equidistance between the length axis and each of the laterals. This process was carried out for each individual handaxe in the study, as well as for the Olduvai choppers and discoids. For these cores, artefacts were oriented along their longest axis, and the most prominent inflection point defined as the "tip" for rotation. If both orthogonal axes were of a similar length, preference was given to the axis with the most heavily worked "tip" for definition as the length axis.

For the side-view images, it was considered important to correct for the lateral orientation of the artefacts, as they often did not lay according to their natural thickness measurement axis. Thus, images were opened in ImageJ 1.52Q, and the line tool used to connect the longest line between the tip and the butt (corresponding to the length axis). The 'Measure' function was then used to output the angle of this line. The deviation of this value from horizontal (i.e. 180°) was recorded. The same image was then opened in GIMP-2.10, and the image rotated about its centre by this deviation, so that the length axis became horizontal. This was repeated for all artefacts for which side-view images were available.

### 2.2. Silhouette Creation

In preparation for landmarking, all plan-view photos were converted into black silhouettes on a white background in GIMP-2.10. This process was not carried out for the side-view images, which were only used to generate thickness measurements and not subjected to geometric morphometric analysis. This was because the large number of images that had to be processed in plan-view left little time to repeat this process for the side-view, although this is something that can be carried out in future analyses.

The silhouettes were then cropped to bring the edges of the image as close to the edges of the handaxe as possible, before all handaxe silhouettes were resized to a 600x400 pixel resolution to try and control for the differences of resolution between different samples.

### 2.3. FlipTest

Symmetry was assessed for the handaxe silhouettes using the FlipTest (Hardaker and Dunn, 2005), without further rotation. This method identifies the midline of an object and flips each lateral so that it overlies the other side. The area for each lateral that does not fall within the boundary of the lateral flipped from the other side is then calculated (asymmetrical pixel count). This is then standardised into an index of asymmetry using the formula:

$$\text{Asymmetry Index} = (500 * \text{Asymmetrical Pixel Count}) / (\text{Maximum Width} + \text{Maximum Height})^2$$

This was carried out twice for each handaxe, once with the tip oriented to the right of the image, and another with the handaxe oriented towards the top of the image. The former provided an estimate of asymmetry about the width axis (latitudinal asymmetry), and the latter provided an estimate of asymmetry about the length axis (longitudinal asymmetry). Both of these metrics have been considered important for understanding the cognition behind handaxe manufacture (Iovita et al., 2017).

2.3. Measurement

A total of four traditional morphometric measurements (defined by Roe, 1968) were taken for each handaxe in imageJ 1.52: length, tip length, width, and thickness. In order to maximise intra-observer reliability for measurements, the standardised orientations were utilised for measurements, meaning they were always exactly orthogonal to one another. In this way, length was calculated for plan view images by the horizontal distance from the tip to the base of the handaxe, but not necessarily to the furthest possible distance from the tip. This avoided ambiguity in determining which point of the artefact was furthest from the tip, and what angle of deviation from horizontal would be acceptable. Width and thickness were calculated from plan- and side-view images, respectively, by the maximum distance from the top and the bottom of the artefact that was orthogonal to the length axis. This avoided many of the same pitfalls as the length measurement, and the orthogonal nature of the measurements avoided any possible redundancies that may have emerged by slightly encroaching upon the other axes. Side-view photos were not available for Kharga Oasis, Olorgesailie, Isinya, and for some individual artefacts in the drawn sample. As such, thickness measurements could not be taken for some specimens, but these could be replaced by caliper measurements in the case of Olorgesailie and Isinya.

Maximum length (from the tip), width, and thickness within 10° of their orthogonal axes were also calculated for artefacts with caliper measurements, as it was suggested that such a measurement may correspond more directly to handheld measurements. These measurements were taken using inflection points in the outline as the necessary start and end of the lines (necessarily the tip for the start of length measurements). Some of this information allowed for correction of the base measurement data, as discussed in the next section.

## 3. Measurement Correction

Three sources of data include caliper measurements that allow comparison to the measurements taken through ImageJ 1.52Q. The Nadaouiyeh handaxes, photographed specifically for this study, could be associated with a series of measurements for each artefact published as supplementary information to Jagher (2016). As such, this sample of 180 handaxes provided an opportunity to identify measurement error associated with the photographic procedure of section 3.1.2. Caliper measurements were also available for handaxe images downloaded from the Marshall et al. (2002) database, and were also collected by CS for the Olorgesailie and Isinya dataset in Shipton (2018). Measurement discrepancies were investigated for each data source individually. All tests are for paired samples, with parametric *t*-tests only used when normality was indicated by a Shapiro-Wilks Test.

*Nadaouiyeh Sample*

With regards to the Nadaouiyeh sample, maximum length, width, and thickness were calculated, in addition to the normal measurements, for each individual artefact. For each of these artefacts, the deviation between caliper and ImageJ measurements was calculated, and artefacts lying more than 1.5x the interquartile range above the upper quartile were removed from the analysis, to avoid correlation of any images to the incorrect artefact in the Jagher (2016) database. This left a sample of 167 handaxes with both length measurements available, 167 with both width measurements, and 148 with both thickness measurements. These larger samples were used to identify the relationship between different measurement variables, and the extent of systematic error. However, of those handaxes remaining with all three measurements, 30 were randomly selected and removed from the dataset using https://www.random.org (Haahr, 2020), with the intention of using these artefacts to cross-validate later measurement corrections. This was a test of how appropriate it may be to apply the regression formulae to the remaining handaxes photographed for this study.

Along the length axis, maximum length (i.e. from the tip to the furthest point on the base within 10° of horizontal) correlated with caliper length marginally more strongly than the baseline length (i.e. exactly horizontal from the tip to the base) measurement, but the difference was negligible ($r$ = .989 vs. $r$ = .988). However, both of these measurements proved to be an overestimate of the caliper length, with maximum length averaging 3.623mm more and baseline length 2.280mm more. Maximum length was selected for further investigation due to its slightly greater correlation with caliper length. Sources of error that may be related to difference between the variables includes lens distortion, the position of the photographic scale, and the resting angle at which the handaxes laid when the plan-view images were taken.

Iovita (2009, cited by Iovita and McPherron, 2011) suggests that lens distortion can be identified in a sample by a correlation between the difference between computer and caliper measurements and the overall size of the object. In this context, this can be best tested by a correlation between the ImageJ and caliper lengths and the caliper length of the object. This analysis does reveal a significant correlation ($r$ = .326, $p$ < 0.001), but it disappears when controlling for caliper thickness in a partial correlation ($r_{partial}$ = .135, $p$ = 0.083). This suggests that the deviation of ImageJ length overestimations was mostly a

product of the vertical distance between the horizontal midline of the handaxe (side-view) and the photographic scale. As such, a regression model using ImageJ thickness and resting horizontal angle of the handaxe (measured in side-view) was used to predict this error in maximum length for each artefact in SPSS 26 (IBM Corp, 2019). ImageJ thickness had to be selected over caliper thickness to have applicability to the other handaxe images taken in the same way, for which caliper measurements were not available. Both variables were found to account for a significant proportion of the variance (F = 33.772, $p < 0.001$) in the difference between ImageJ and caliper measurements (Model $R^2_{adj.} = .325$). The corresponding regression formula was:

ImageJ Maximum Length – Caliper Length = 0.047 + 0.185*ImageJ Thickness – 0.176*Horizontal Angle

Subsequent application of this formula to the maximum length measurements of the 30 handaxes kept to the side at the beginning of the process resulted in no significant difference to the caliper measurements ($t_{paired} = 1.284$, $p = .209$). This new variable was denoted “Corrected Length”. As such, it was deemed acceptable to apply this formula to measurements on the same axis in the wider dataset (tip length and baseline length). The baseline length correction was used as the main length variable throughout the investigation.

With regards to width, baseline width (i.e. longest measurement between the laterals orthogonal to the length axis) was more strongly correlated with caliper width than was maximum width (i.e. longest measurement between the laterals within 10° of orthogonal to the length axis; $r = .981$ vs $r = .964$). As such, only baseline width is discussed from this point onwards. Again, however, the ImageJ width overestimated measurements taken by calipers by 2.334mm. While resting angle in side-view is unlikely to have impacted plan-view width measurements (because they belong to different axes), resting angle in front view may have had an impact but this information was not available. The other potential sources of error match that of the length axis, namely lens distortion and position of the photographic scale. However, unlike for the length measurements, the correlation of the difference between ImageJ and caliper width to the caliper length ($r = .282$, $p < 0.001$) does not disappear when controlling for caliper thickness, it is only weakened ($r_{partial} = .190$, $p = 0.014$). In contrast, while there is a relationship between the difference in width measurements and caliper thickness ($r = .184$, $p = 0.013$), this is removed when controlling for length ($r_{partial} = .113$, $p = 0.148$). Taken together, this suggests lens distortion may have influenced width measurements moreso than length measurements.

Given the results of the correlation analyses, the new corrected ImageJ length variable (as the closest computerised proxy for caliper length) was deemed most suitable variable to attempt to correct for the ImageJ width measurements. Indeed, it was put into a regression model in SPSS 26 to predict differences between ImageJ width and caliper width, and explained a significant (F = 17.232, $p < 0.001$) proportion of the variance ($R^2_{adj.} = .107$). The regression formula was output as:

ImageJ Width – Caliper Width = -.149 + 0.028*Corrected Length

This formula was used to correct ImageJ width measurements of the 30 handaxes not used in model formation. As with length, the resulting values were not significantly different to the caliper width measurements ($t_{paired}$ = .178, $p$ = 0.860), and thus the formula was used to correct width measurements for all other handaxe images taken by the same method.

Baseline thickness measurements in ImageJ (i.e. longest line from the top to the bottom of the handaxe in lateral view, orthogonal to the length axis) were also more strongly correlated with caliper thickness than maximum thickness (i.e. longest line from top to bottom within 10° of orthogonal to the length axis; $r$ = .964 vs. $r$ = .962). Nonetheless, in contrast to length and width, baseline ImageJ thickness actually underestimated caliper thickness by an average of 2.926mm. Lens distortion has not had a significant impact on this outcome, as the correlation for the difference between ImageJ and caliper thickness with caliper length does not reach significance ($r$ = .177, $p$ = 0.055). Instead, by far the most likely explanation for the underestimate, is that the photographic scale was placed on a horizontal plane in front the handaxe for the side-view photos, in contrast to the plan-view images where it was behind (below). An attempt to investigate this was carried out by correlating the difference between ImageJ and caliper thickness measurements with the caliper width of the handaxe (as a proxy for distance between the photographic scale and the midline of the handaxe), but the results were insignificant ($r$ = -.074, $p$ = 0.427). As such, the positioning of the scale may have added a more-or-less constant source of error to the measurements, that did not, in itself, covary with the size of the handaxe. However, this difference may have been exacerbated amongst thinner handaxes, as the deviation between ImageJ and caliper thickness is significantly negatively correlated with actual caliper thickness ($r$ = -.505, $p$ < 0.001).

To correct for this source of error, ImageJ thickness was used as the sole independent variable in an SPSS 26 regression analysis to predict the difference between ImageJ and caliper thickness measurements. Again, this accounted for a significant (F = 16.958, $p$ < 0.001) proportion of the variance ($R^2$ = .120), and application of the regression formula to the left-out 30 handaxes removed any difference to caliper measurement ($z$-transformed Wilcoxon Signed Ranks Test: $z$ = -1.121, $p$ = 0.262). The regression formula is as follows:

ImageJ Thickness – Caliper Thickness = -1.041 – 0.068*ImageJ Thickness

As the formula removed discrepancy between ImageJ and caliper measurement, it was used to correct the thickness measurements for the remaining samples for which images were taken in the same way.

*Marshall et al. (2002) Sample*

For the samples derived from the Marshall et al. (2002) database, maximum length, width, and thickness measurements were taken for the first five handaxes from each assemblage included from this source, resulting in a total sample of 65 handaxes. This was done to maximise the variability with which measurements could be evaluated. As with the Nadaouiyeh samples, a variable of difference between ImageJ and caliper measurements was computed, and values lying more than 1.5x the interquartile range above the upper quartile were removed. This left a sample of 58 length measurements, 59 width

measurements, and 62 thickness measurements. Initial correlations of measurements were done with the entire sample, but a subsample of 25 handaxes was randomly set aside from regression models for subsequent cross-validation, using https://www.random.org (Haahr 2020).

Amongst the entire Marshall et al. (2002) subsample, maximum ImageJ length was marginally more strongly correlated with caliper length than baseline ImageJ length, but the difference is extremely minor ($r$ = .9986 vs $r$ = .9991). However, despite these extremely high correlations, both ImageJ length measurements present an underestimate compared to the caliper length (average of 2.105mm for baseline and 0.8391mm for maximum). This source of error was investigated using ImageJ maximum length, owing to its greater correlation with caliper length, and for consistency with the Nadaouiyeh sample. Despite the difference of <1mm, maximum ImageJ length is significantly different from caliper length ($z$-transformed Wilcoxon Signed-Ranks Test: $z$ = -3.620, $p$ < 0.001). Unlike for the Nadaouiyeh sample, the angle at which the handaxe rested in side-view did not covary with the difference between ImageJ length and caliper length ($r$ = .110, $p$ = .412). Furthermore, the difference between ImageJ maximum length and caliper length was correlated with the caliper length of the artefacts ($r$ = -.292, $p$ = 0.026), and this could not be controlled by other handaxe dimensions, suggesting a possible weak effect of lens distortion.

In order to try and control for this source of error, ImageJ maximum length was used as the sole independent variable in a regression model in SPSS 26, attempting to predict differences between ImageJ maximum length and caliper length. The random sample of 25 handaxes was excluded for this. However, this model did not reach significance (F = 1.196, $p$ = .283), meaning no regression formula could be used to correct for the lens distortion. It is worth noting that this effect was still weak and could not explain a large proportion of the difference between ImageJ and caliper measurements. We would suggest that there was thus a source of constant error in the ImageJ measurements, perhaps related to distortion of the scale in the low-resolution images, resulting from initial rotation of the images. This process tended to blur the edges of the scale, and thus setting the scale by its edges may have actually been utilising a previous position of the scale's extremities, overestimating the length of a given distance and thus underestimating the measurements by a constant amount. Thus, while not ideal, the only way deemed possible to correct for the error in length data was to subtract the mean difference between ImageJ maximum length and caliper length for the subsample as a whole (-0.839138) from the ImageJ length axis data. It was not deemed worthwhile to calculate this difference from a subset of the subsample and attempt to judge generalisability to a second subsample, because a better mean estimate for the difference would be given for the larger sample size.

Caliper width from the Marshall et al. (2002) database is significantly correlated with both baseline ImageJ width and maximum ImageJ width, although it is marginally stronger for the latter ($r$ = .996 vs. $r$ = .997). In accordance with the length data, both width measurements present underestimates compared to the caliper measurement, by an average of 1.809mm for baseline width and 0.839 for maximum width. Despite the slightly higher correlation for maximum ImageJ width, baseline ImageJ width is taken here to investigate discrepancy to caliper data for consistency of measurement with the Nadaouiyeh sample. Had this decision not been taken, the Marshall et al. (2002) handaxes would likely have had width

measurements systematically greater than the handaxes photographed for this study because of a less severe corrective procedure. As such, the baseline ImageJ width measurements were found to be significantly lower than the caliper measurements (*z*-transformed Wilcoxon Signed-Ranks Test: $z = -6.079$, $p < 0.001$).

The difference between ImageJ width and caliper width is significantly correlated with caliper length ($r = -.294$, $p = .033$) and caliper width ($r = -.357$, $p = .006$). Furthermore, while the former correlation disappears when controlling for caliper width ($r_{partial} = .115$, $p = .415$), the latter correlation persists when controlling for caliper length ($r_{partial} = -.287$, $p = .039$). These relationships are interpreted as a minor effect of lens distortion, as detected for the length axis, but there is also a slightly stronger effect that is unique to the width axis. This may be related to the resting angle of the handaxe in front view, but again this data is not available to test. Nonetheless, ImageJ width was subsequently used in a regression analysis in SPSS 26 to try and predict the discrepancy between ImageJ width and caliper width, with the random sample of 25 handaxes excluded from the investigation. Unfortunately, this model also did not reach significance ($F = 2.117$, $p = .155$), and thus the minor effects of lens distortion and the width-axis-specific source of error could not be corrected for. Nevertheless, these measurements suffered from the same issues of scale as the length axis, and thus it was deemed valid to subtract the mean difference between baseline ImageJ width and caliper width across the whole subsample (-1.808475) from the baseline width. Again, for the same reasons as the length data, it was not deemed appropriate to compute this step for a subset of the subsample.

Finally, the caliper thickness measurements are slightly more closely related to baseline thickness in ImageJ than to maximum thickness in the whole subsample ($r = .998$ vs. $r = .997$). Baseline thickness again represents an underestimate compared to the caliper measurements (average of 1.049mm. This difference was highly significant (*z*-transformed Wilcoxon Signed Ranks Test: $z = -6.366$, $p < .001$). This difference, and its similar magnitude to the length axis, may again support the presence of a constant source of error associated with scale. The larger error associated with the width related to both this effect and the larger unknown source of width-specific error. Nonetheless, the difference between ImageJ baseline thickness and caliper thickness is significantly correlated with all caliper dimensions of the handaxe at $\alpha = 0.01$. This is strongest for width ($r = -.429$, $p = .001$) and thickness ($r = -.415$, $p = .001$). The effects are not restricted to one axis in particular, as the correlation for width disappears when controlling for caliper thickness ($r_{partial} = -.263$, $p = .153$), and the correlation for thickness disappears when controlling for caliper width ($r_{partial} = -.266$, $p = .149$). This suggests a relatively stronger impact of lens distortion on the side-view handaxe images from the Marshall et al. (2002) database, with the reasons for this unclear. There is no evidence of any other variable impacting the discrepancy between ImageJ and caliper thickness.

As width and thickness were covaried most strongly with the difference between ImageJ and caliper thickness measurements, ImageJ width and ImageJ thickness were taken as independent variables in a stepwise regression analysis in SPSS 26 to predict the discrepancy. The 25 randomly-selected artefacts were excluded from this analysis. ImageJ thickness was ultimately excluded by the program because its covariance was mostly accounted for by width, as predicted by the partial correlation. Proceeding with ImageJ

width as the only input variable, the regression model did reach significance (F = 8.534, $p$ = .006), with an $R^2_{adj.}$ value of .169. The output regression formula is given by:

$$\text{ImageJ Thickness} - \text{Caliper Thickness} = 0.304 - 0.20 * \text{ImageJ Width}$$

This formula was used to calculate the estimated difference between the thickness variables for the 25 artefacts not used in model creation. When this difference was subtracted from ImageJ thickness to create a Corrected Thickness variable, mean difference compared to caliper width had completely disappeared ($t_{paired}$ = -.530, $p$ = .601). As a result, this procedure was applied to the thickness measurements for the remaining handaxes from the Marshall et al. (2002) sample.

*Olorgesailie and Isinya Sample*

For the Olorgesailie and Isinya material, lateral-view images were not available, and thus computer-derived thickness measurements could not be taken. Thus, caliper thickness was used for morphometric ratios requiring thickness (i.e. refinement). However, thickness measurements were removed from artefacts for which both length and width differences were found to be outliers, which was the case for 2 specimens. This left an initial 42 specimens for the analysis. Maximum length and width measurements (within 10° of their respective axis) were taken in ImageJ for all specimens in this sample.

Regression analyses were carried out to try and correct for sources of error in both length and width measurements. For both length and width, the difference between the caliper and ImageJ measurements were calculated, and outliers (more than 1.5x the interquartile range above the upper quartile) removed from each measurement sample. However, no artefacts were removed from the analysis for later cross-validation, because the results would not be extrapolated onto another part of the dataset, and only themselves need correcting.

Caliper length was fractionally more strongly associated with the ImageJ maximum length variable than for baseline length ($r$ = .99795 vs. $r$ = .99792). While there is no real difference between these variables, maximum length was again taken for further investigation for consistency. This variable produced a drastic overestimate of caliper length, by an average of some 11.8mm. The difference between ImageJ maximum length and caliper length was also strongly correlated with each of the three main caliper measurements, with $r$-values of .762 ($p < 0.001$), .742 ($p < 0.001$), and .611 ($p < 0.001$) for length, width, and thickness respectively. Unlike in the Nadaouiyeh sample, the effect of length cannot be accounted for by caliper thickness ($r_{partial}$ = .609, $p < 0.001$), suggesting the effect is not simply caused by vertical distance from the photographic scale. These relationships suggest a much stronger effect of lens distortion than for the other sources of data, and suggest the strength of this effect is increased for larger measurements.

As caliper length covaried with the difference between ImageJ maximum length and caliper length most strongly, ImageJ maximum length was used as the sole input into a regression model in SPSS 26 to correct for the disparity. This model performed extremely well (F = 66.8,

$p < .001$) in explaining the differences between ImageJ maximum length and caliper length ($R^2_{adj.}$ = .634), outputting a regression formula of:

$$\text{ImageJ Maximum Length} - \text{Caliper Length} = -1.906 + 0.079 * \text{ImageJ Maximum Length}$$

This formula was applied to the dataset used in model creation to calculate a new corrected length variable, and this inevitably removed the differences between ImageJ and caliper measurement ($t$ = -.119, $p$ = 0.906). As such, this formula was also used to correct baseline length and tip length.

ImageJ baseline width was more strongly correlated with caliper width than was ImageJ maximum width ($r$ = .991 vs $r$ = .987). Baseline width presented an overestimate for caliper length of 4.678mm, further supporting the argument that differences between ImageJ and caliper measurements are lesser for smaller measurements. This pattern is also identified in the significant correlations between the ImageJ and caliper differences and caliper measurements, whereby the respective $r$-values of .487, .489, and .470 for length, width, and thickness are smaller than for the length axis. As a result of these coefficients, ImageJ width was taken as the sole independent variable in a regression analysis in SPSS 26. This model was also significant (F = 21.961, $p < .001$), with an $R^2_{adj.}$ value of .344. The model produced a regression formula of:

$$\text{ImageJ Width} - \text{Caliper Width} = -3.613 + 0.091 * \text{ImageJ Width}$$

This formula was used to produce a corrected width variable for the same sample, again removing the difference between ImageJ and caliper measurement ($t_{paired}$ = -.091, $p$ = 0.928). Accordingly, this new width variable was taken as the width measurement for these handaxes.

*Summary*

To summarise, a small amount of systematic measurement error is present in all of the sources of data, but steps have been taken to correct these issues. Overall, not a great deal of this error amongst images taken according to the procedure in section 3.1.2. can be attributed to lens distortion having a systematic impact on the measurements. Instead, most identifiable error derives from the position of the photographic scale and the resting angle of the handaxe in side-view. There may be some evidence of lens distortion in the measurement of width. In contrast, lens distortion is discernible on all three axes for the Marshall et al. (2002) photographs, particularly for the side-view images, potentially owing to the comparatively poorer quality of photographic equipment when the database was compiled. The other notable source of error in this sample derives from the distortion of the scale when rotating the low-resolution images. Finally, the Olorgesailie and Isinya handaxe images show the clearest impact of lens distortion, with the reason for this not immediately clear given the recency of their capture. One potential explanation is that distortion is exacerbated by the increased raw size of the artefacts compared with those in the other samples.

Taken together, lens distortion is a possible confounding variable for the geometric morphometric analysis. Nonetheless, it is relatively minor in magnitude for a large majority of plan-view handaxe samples (collected for this study and from the Marshall et al. [2002] database), and these are distributed throughout the study regions. Thus, these handaxes are unlikely to have affected one region more than the other. In contrast, lens distortion is stronger amongst the Olorgesailie and Isinya photographs, and this is restricted to one individual region. While this source of error is acknowledged, these localities form a relatively small part of the African sample and thus is not expected to have rendered the data uninformative.

Finally, it is important to acknowledge that it was not possible to test the validity of (and therefore correct for) measurements taken for the 2D images deriving from 3D scans for Moulin Quignon and Gesher Benot Ya'aqov, and for the drawings of Olduvai, Kalambo Falls and the Kapthurin Formation. However, lens distortion is very unlikely to result from 3D scans, whereby error in individual components will be corrected by the relationship to other components of the model, while the drawings were photographed according to the procedure in section 3.1.2, that we have already seen has not resulted in extensive distortion. The most likely source of error in the drawings derives from the bending of pages as the photograph was taken but, while this cannot be corrected for, every effort was taken to minimise this effect. Kharga Oasis plan-view images were taken according to a procedure similar to that outlined in section 3.1.2, despite not having side-view images, and thus were corrected according to the length and width axes formulae derived from the Nadaouiyeh handaxes.

**Supplementary Information 3: Site Descriptions**

## 1. African Sites

### 1.1. Olduvai Gorge

Olduvai Gorge, Northern Tanzania, is a valley incised by an extinct river, previously flowing from Lakes Masek and Ndutu to the west of the Olduvai basin (Hay, 1976; Tamrat et al., 1995). The depositional sequence of the site spans some ~100m in thickness, split into seven main sedimentary units: Beds I-IV, the Masek Beds, the Ndutu Beds, and the Naisiusiu Beds (Hay, 1976). Stratigraphic subdivisions in the basin are related to the position of marker tuffs (primary volcaniclastic deposits and reworked volcaniclastic sandstones; McHenry and Stanistreet, 2018) and major disconformities between sedimentary units (Hay, 1976; Njau et al., 2020; Stanistreet et al., 2020). The main Acheulean assemblages, including those studied here, are found at numerous sites from within Beds II-IV, and as such these units form the basis of subsequent discussion about the site. Each of these three units are associated with remains of Homo erectus, with Homo habilis and Paranthropus boisei specimens also present in Bed II (Domínguez-Rodrigo et al., 2013; Leakey, 1971).

The Olduvai Gorge Coring Project (OGCP) has produced four cores drilled into the sequence, that give a detailed and continuous picture of deposition in the basin over time (Stanistreet et al., 2020). Modes of deposition in the Olduvai sequence are varied, encompassing lacustrine, fluvial, alluvial, aeolian, volcaniclastic, and tuffaceous sediments (Hay, 1976; Njau et al., 2020; Tamrat et al., 1995). This is, perhaps, exemplified by Bed II, in which tuffs are relatively abundant and interspersed within sediments from Palaeolake Olduvai, alluvial fan debris, and a fluvio-deltaic system (McHenry and Stanistreet, 2018; Stanistreet et al., 2020). This abundance of dateable tuffs has been very important for establishing the chronology of Bed II. It lies directly (and comfortably) upon Tuff IF, at the very top of Bed I, which has provided a maximum age of 1.803 ± 0.002 Ma for all of the bed (Deino, 2012). The OGCP age model based on 40Ar/39Ar dates and tephrostratigraphic correlations places the end of Bed II at 1.14 ± 0.05 Ma (Deino et al., 2021).

The Bed II sites included in the present study are EF-HR, TK, and BK. EF-HR was originally correlated to upper Middle Bed II by Leakey (1971), underneath Tuff IIC which delineates the boundary between the Middle and Upper parts of the bed. However, McHenry and Stanistreet (2018) report that the archaeological levels at the site lie on a major unconformity that, to the west, cuts through Tuff IIC, suggesting the site is above this marker horizon in Upper Bed II. While Tuff IIC is not, currently, well-dated, the site must be younger than the age of 1.664 ± 0.019 Ma given by Diez-Martín et al. (2015) for above the Middle Bed II site of FLK-W, one of the oldest Acheulean sites in the basin (McHenry and Stanistreet, 2018). Furthermore, the archaeological levels at EF-HR clearly lie underneath Tuff IID, another marker tuff in Bed II, which is dated to 1.338 ± 0.024 Ma (Domínguez-Rodrigo et al., 2013), providing a minimum age constraint. Sites TK and BK both lie just above Tuff IID, meaning they can be bracketed between 1.338 ± 0.024 Ma and the end of the bed at 1.14 ± 0.05 Ma (Deino et al., 2021; Domínguez-Rodrigo et al., 2009, 2013). Leakey

(1971) correlates BK with a slightly higher position in Bed II than TK, suggesting it may be the younger of the two sites.

Nonetheless, Beds I-IV are all dominated by a claystone and sandy claystone lithology that relates to lacustrine and lake-shore deposition by Palaeolake Olduvai (Stanistreet et al., 2020). Fluctuations in lake level appear to increase in severity over time (both seasonal and longer-scale), and there is an overall trend towards reduced lake depth from Beds I-IV. Indeed, in Bed IV times, there were at least three sustained periods of low lake level, in which the river incised into the lakebed, resulting in conglomerate formation and deposition of fluvial sands and gravels (Stanistreet et al., 2020). Malacofauna suggest a similar pattern for Bed III, as the species indicate a periodically desiccated floodplain bordering a perennial river channel(s), with marked seasonal variation (Johnson et al., 2016). That processes were similar between Bed III and IV is attested to by the fact that the sedimentology of the units is identical in many areas of the gorge, except in the eastern part of the basin, where a braided stream system in Bed III led to coarser-grained input (Hay, 1976; Njau et al., 2020; Stanistreet et al., 2020; Tamrat et al., 1995).

However, the chronology of the Olduvai sequence has, until recently, been much more tightly constrained for the lower two beds than for the upper beds. This can be attributed to only discontinuous exposures of the upper beds within the basin, their relative lack of tuffaceous marker horizons and the frequent erosive contacts between units and subunits (Njau et al., 2020). Furthermore, taphonomic data suggests poorer preservation of archaeological material in the upper beds due to weathering and erosion (Leakey and Roe, 1994), resulting in a lack of material available for direct dating and a research bias towards the lower beds (Njau et al., 2020). As such, previous dating attempts for Beds III and IV have derived from the positioning of palaeomagnetic shifts and corresponding estimates for the rate of sedimentation (Hay, 1976; Tamrat et al., 1995; Walter et al., 1992). Hay (1976) suggested the beginning of the Brunhes chron (773 ka) began within the Bed IV sequence, and suggested an age of 830-600 ka. In contrast, Tamrat et al. (1995) identified 3 periods of reversed (R1-R3) and 2 periods of normal (N1-N2) polarity in the sequence from Bed I to the Masek Beds at Olduvai. N1 is clearly correlated with the Olduvai subchron (1.93-1.78 Ma) by 40Ar/39Ar dates of tuffs from Upper Bed I and Lower Bed II, leaving N2 (at the boundary between Bed IV and the Masek Beds) as either the Jaramillo subchron (1.07-0.99 Ma) or the onset of the Brunhes chron (Tamrat et al., 1995). As the authors detected another reversed zone after N2, they suggest that N2 represents the Jaramillo normal phase within the Matuyama chron, suggesting all of Beds I-IV were deposited before 1.01 Ma (Tamrat et al., 1995).

This debate can be settled using the comprehensive age model produced by the OGCP, incorporating new palaeomagnetic, 40Ar/39Ar, and tephrostratigraphic data (Deino, 2012; Deino et al., 2021; Stanistreet et al., 2020). This work places the base of the Jaramillo subchron in Bed III, and the base of the Brunhes in the Masek beds, confirming that Beds I-IV were all deposited before 773 ka (Deino et al., 2021). Furthermore, tephrostratigraphic correlations combined with old and new 40Ar/39Ar dates completely bracket Bed III between 1.14 ± 0.05 Ma and 0.93 ± 0.08 Ma, and Bed IV between 0.93 ± 0.08 and 0.82 ± 0.06 Ma (Deino et al., 2021). The Bed III sample used in this investigation derives from throughout the unit and thus may fall anywhere between the Bed III ages. Bed IV samples

were taken from sites HK, MK, and MNK, but it is not possible to arrange these into a stratigraphic succession. Artefacts derive from L.S.B. Leakey's (1931-1934) excavations, from which he describes artefacts from throughout Bed IV at HK, and very little description is given to MK (Leakey, 1951). The MK artefacts are described as deriving from an intermediate stratigraphic position in Bed IV, but this must be treated with extreme caution given the numerous disconformities now recognised in the Bed IV sequence (Njau et al., 2020). As such, I do not attempt to make a finer-scale chronological distinction between the assemblages than the current age brackets for Bed IV.

Leakey (1971) describes the archaeological succession between the Oldowan (Bed I and Lower Bed II) and the Acheulean as a gradual process, in which the "Developed Oldowan A" (DOA) and the "Developed Oldowan B" (DOB) represent transitional Bed II phases before the appearance of the Acheulean, respectively below and above Tuff IIB. The DOA was characterised by mostly Oldowan artefacts, but a higher frequency of spheroids and subspheroids, and light-duty tools, while the DOB was presented as very similar to the Acheulean, but with smaller handaxes that make up <40% of the assemblage (Leakey, 1971). Leakey and Roe (1994) also suggested that the Developled Oldowan persisted into Bed IV, with the Developed Oldowan C (DOC). However, it is now thought that the DOA and DOB may be functional facies of the Oldowan and Acheulean (e.g. Semaw et al., 2009), in which the DOA represents increased investment in technology through existing Oldowan techniques (e.g. Proffitt, 2018) and the DOB represents Acheulean sites with differences in handaxe discard patterns or ecological function (e.g. Gowlett, 1988; Hay, 1976, 1990; Isaac, 1969; Pope and Roberts, 2005; Potts, 1989; Uribelarrea et al., 2017; Sánchez-Yustos et al., 2019). Indeed, DOA, DOB, and Acheulean assemblages are interstratified at FLK-W, suggesting there is no linear progression between them (Sánchez-Yustos et al., 2018).

The Olduvai samples included in the study derive from different sources. The Bed II handaxe data was extracted from M. Leakey's (1971) monograph on excavations in Beds I and II at Olduvai. This produced a total of 36 Bed II handaxes, 14 of which derive from EF-HR, 8 come from TK, and 14 come from BK. Amongst the Bed II handaxes, all are described as being made on lava or quartz/quartzite by Leakey (1971). The 27 Bed III handaxes, made on quartzite and a variety of lavas (basalt, andesite, nephelinite, and andesite) come from a selection of different sites, and were selected from the Marshall et al. (2002) biface database. The same database was also used for the Bed IV samples, which numbered 30 artefacts from HK, 16 from MK, and 18 from MNK. These handaxes have the same raw materials as for Bed III, but show an overwhelming shift towards use of quartzite, as also suggested by Shipton (2018).

### 1.2. Olorgesailie

The Olorgesailie Formation, southern Kenya, is an extended (~80m) sequence of Early and Middle Pleistocene sediments, deposited in a lake basin delineated to the south by Mount Olorgesailie and Mount Shanumu, and to the east and west by plateau lavas (Isaac, 1977; Potts et al., 1999). The formation consists of 14 members, beginning with Member 1 at the base, variously comprising of lacustrine, lake margin, fluvial, and fluvio-deltaic sediments (Deino and Potts, 1990; Owen et al., 2008). Diatoms are also an important component of the sedimentary units, and have allowed palaeoenvironmental reconstruction over time,

indicating large fluctuations in lake level, alkalinity, and salinity over time, in addition to lake-marginal wetlands and terrestrial palaeosol formation (Owen et al., 2008).

The three assemblages from Olorgesailie included in the present study derive from sites CL1-1 (lower Member 1), I3 (upper Member 1 palaeosol; UM1p), and multiple sites from the diatomaceous silts and sands of Member 6 and lower Member 7 (Potts et al., 1999; Shipton, 2018). CL1-1 is bracketed, somewhat widely, by an 40Ar/39Ar date of ~1.2 Ma (full age not reported; Owen et al., 2008) below the archaeological horizon and towards the base of Member 1, and above by a weighted mean age of 992 ± 39 ka for the middle of the member (Deino and Potts, 1990). The occupation at CL1-1 took place in the wider context of a mildly-saline, silica-rich water body in the Olorgesailie landscape, derived from a spring (Owen et al., 2008). The age of 992 ± 39 ka for middle Member 1 also provides a maximum age for the assemblage at I3, formed on a palaeosol at the top of the member, and a period of alternating freshwater and moderate-to-highly saline water bodies (Owen et al., 2008). A minimum age can be derived from 40Ar/39Ar dates for Members 4 and 5, of 960 ± 16 ka and 974 ± 7 ka, respectively (Deino and Potts, 1990).

Member 6/7 represents another period of terrestrial palaeosol formation, crossed by multiple fluvial channels, and is also associated with the calvarium of a small-brained (<800 $cm^3$) Homo erectus specimen (Owen et al., 2008; Potts et al., 1999, 2004). The dates for Members 4 and 5 tightly constrain a maximum age, and a minimum age of 747 ± 6 ka is given by the next 40Ar/39Ar date in the sequence, towards the top of Member 8 (Deino and Potts, 1990; Potts et al., 2004). Chronostratigraphic evidence would suggest the unit dates closer to the upper age limit, given there is no unconformity between Member 5 and Members 6/7, but there may be a hiatus before the start of, or possibly during, Member 8 deposition (Durkee and Brown, 2014; Potts et al., 2004). This observation can be evaluated by the palaeomagnetic evidence. Firstly, the Brunhes-Matuyama reversal can be clearly identified near the middle of Member 8, providing a slightly higher minimum age of 773 ka for Members 6/7 (Durkee and Brown, 2014; Tauxe et al., 1992). Secondly, Tauxe et al. (1992) also detected two very short normal intervals within the Matuyama chron, one in Member 7 (above Member 6/7) and another in lower Member 8. According to Durkee and Brown (2014), there are only three possible normal polarity events that could account for this pattern, namely the Santa Rosa Event at 932 ka, the Kamikatsura Event at 906 ka, or a normal event immediately before the onset of the Brunhes chron, dated to 776 ± 2 ka in Maui (Coe et al., 2004; Singer, 2014). As such, the older of the two normal events at Olorgesailie must either represent the Santa Rosa or Kamikatsura, meaning Member 6/7 cannot be younger than 906 ka (Durkee and Brown, 2014).

The archaeological assemblages at Olorgesailie are known for the extremely dense concentrations of bifaces and faunal remains at specific sites, as well as the patterns of landscape use carried out by hominins at the site (Isaac, 1977; Potts, 1989; Potts et al., 1999; Shipton, 2018). This includes a pattern in which bifaces are very rarely identified at lake margin sites, but instead accumulate near raw material sources and channel contexts, in spite of flakes being present that are clearly from handaxe reduction (Potts, 1989; Potts et al., 1999). This may indicate that hominins were producing bifaces at raw material sources, transporting them around the lake basin, and depositing them at the channel pathways between the lowland basin and highland environments (Potts et al., 1999).

Throughout the sequence, most bifaces are made on local lavas, with although with some material transported up to 45km into the site (Isaac, 1977; Potts et al., 1999). Amongst the assemblages included in this study, CL1-1 stands out for a substantially greater proportion of cleavers than handaxes (Shipton, 2018), resulting in its low sample size (n = 5), and its focus on basalt as a raw material. The upper Member 1 and Members 6/7 samples number 15 and 11, respectively, and are both dominated by phonolite. All data was collected by CS at the National Museums of Kenya for Shipton (2018).

## 1.3. Isinya

The Acheulean site of Isinya (formerly Isenya) is located in the Eastern Highlands of southern Kenya. Artefacts derive from the lowermost of five units within the 3m sedimentary sequence (Roche et al., 1988). This unit (Unit I) represents a series of fluvial sediments from a perennial river, with hominins repeatedly active atop a sandy bar deposit, resulting in artefacts and fauna being recurrently buried (Durkee and Brown, 2014; Roche et al., 1988). The faunal remains overwhelming belong to an open savannah environment (Roche et al., 1988).

The only current indication of age for Isinya has been achieved by tephrostratigraphic correlation between tuffs in Unit 3 and other Early Pleistocene Acheulean sites in southern Kenya (Durkee and Brown, 2014). Two samples from the site have extremely similar geochemical composition to a pumice from Member 4 at Olorgesailie, implying that the tuffs were formed by the same volcanic event. This member at Olorgesailie has been 40Ar/39Ar dated to 960 ± 16 ka (Deino and Potts, 1990). While this gives a minimum age for the artefacts at the site, the maximum age of the deposits is unknown because the Kapiti Phonolite (also the main source of raw material) on which the sediments lie derives from the Miocene (Durkee and Brown, 2014; Roche et al., 1988).

The site has produced numerous bifaces from the seven main archaeological layers, and are known for their high technical skill, despite their relatively early chronological position. This characterisation is based on numerous features, including predetermination of flake blank morphology, and early evidence for use of a soft hammer in reduction (Texier, 2018). A total of 22 handaxes were available for study, with data collected by CS at the National Museums of Kenya for Shipton (2018). A large majority (18/22) are produced on local phonolite, with isolated appearances of quartz, chert, and consolidated tuff.

## 1.4. Kariandusi

Kariandusi is located 2km east of Lake Elmenteita in the wider Nakuru-Elmenteita Basin (Gowlett and Crompton, 1994). The 45m sedimentary succession has been exposed by the incision of the River Kariandusi as it travels from the nearby escarpments to Lake Elmenteita. This has allowed identification of two artefact-bearing loci, either side of the gorge: the Upper Site and the Lower Site (Durkee and Brown, 2014; Gowlett and Crompton, 1994; Shipton, 2011). At both sites, all sediments overlie the Gilgil Trachyte and artefacts derive from tuffaceous gravels and finer tuff beds that lie above two basal diatomite units (Durkee and Brown 2014; Gowlett and Crompton, 1994). Palaeoenvironmental

reconstruction suggests that hominin occupation may have taken place at a time when the basin contained several giant lakes, substantially deeper than Lakes Nakuru or Elmenteita today (Gowlett and Crompton, 1994; Nyamweru, 1980; Washbourn, 1967). There is also likely to have been multiple different environmental contexts within a few kilometres of the site (Gowlett and Crompton, 1994).

In contrast to Isinya, several maximum ages are available for the artefacts at Kariandusi. A date of 977 ± 10 ka has been provided for a grey ash layer lying above the upper diatomite unit, but below the artefact horizons (Trauth et al., 2005). In addition, geochemical sourcing of obsidian artefacts suggest the obsidian derives from three sites (10-30km away; Merrick et al., 1994). The formation of one of these sites, the Gilgil Toll Station, has been Potassium-Argon (K-Ar) dated to 970 ± 30 ka (Durkee and Brown, 2014), consistent with the maximum age derived from the section. This age can be refined further when one considers that a bedded lapilli tuff from Kariandusi (also underneath the artefacts) can be correlated with the same tuff at Olorgesailie Member 4 as Isinya (Durkee and Brown, 2014). This confirms that the Kariandusi artefacts are younger than those at Isinya, and are younger than the date of 960 ± 16 ka given by Deino and Potts (1990) for Member 4 at Olorgesailie (Durkee and Brown, 2014). The minimum age of the site is less well-established, but a palaeomagnetic sample from a blue-grey pumice tuff overlying the artefacts at the Lower Site (stratigraphically lower in the sequence than the Upper Site) returned a negative polarity (Gowlett and Crompton, 1994), confirming that all artefacts are confined to the Matuyama Chron and are at least 773 ka.

All artefacts in the present study derive from L.S.B. Leakey's (1931) 1929-1931 excavations at the Upper Site, subsequently exported to the MAA, Cambridge. In addition to obsidian, the handaxes are also frequently made on lavas (local Gilgil Trachyte, semi-exotic rhyolite, and phonolitic basalt; Gowlett and Crompton, 1994; Shipton, 2011). The final sample from the site numbered 27.

### 1.5. Kalambo Falls

The prehistoric sites of Kalambo Falls are located on the Zambian side of the Kalambo River, at the border with Tanzania (Clark, 2001). The sediments at the site are almost completely fluvial in nature, but the absence of a regular terrace stratigraphy has made interpretation and dating more difficult than some of the European sites included in the present study (Duller et al., 2015). Deposition can be described as successive "packages", consisting of coarse-grained sand and gravel channel sediments and finer-grained sand overbank sediments, in the context of meander migration across the floodplain (Barham et al., 2015; Duller et al., 2015). This repeated migration has also heavily truncated the top of successive packages, and these unconformities thus frequently result in lag deposits with high concentrations of artefacts reworked from destroyed sediments (Barham et al., 2015; Duller et al., 2015). That the artefacts are in secondary position is also suggested by their sometimes-vertical orientation but, at least for Acheulean levels, they also remain in a fresh condition and show stratigraphic consistency, suggesting movement may have been limited (Barham et al., 2015; Clark, 2001; Duller et al., 2015; Schick, 2001).

Clark (2001) reports on excavations at four sites, A-D, amongst which the Acheulean deposits are overwheliming located within the basal Mkamba Member (or Package 1 in modern re-excavation) of the sedimentary sequence at Sites A and B (Barham et al., 2015; Clarkm 2001). This frequently takes the form of pebble 'horizons' (now interpreted as reworked aggradations) interstratified within the white sand beds of the Mkamba member (Clark, 2001). The preservation of plant material (particularly wood) due to waterlogging and recovery of pollen has allowed reconstruction of a persistent open floodplain environment, with indications of adjacent swamp forest and woodland (Taylor et al., 2001; Barham et al., 2001). The environment may also have been warmer and drier compared to the present day (Taylor et al., 2001), but this cannot be suggested with great certainty.

The preserved wood from the site has been subject to direct dating methods in the form of AAR and U-Series, with the former giving a minimum age of 110 ka (Lee et al., 1976). The U-Series dates for stratigraphically lower and higher Acheulean levels of 182 ± 16 ka and 182 ± 10 ka, respectively (McKinney, 2001), have been extremely influential in interpreting the site as very young in the context of East African assemblages (e.g. Sahnouni et al., 2013). However, the presence of sufficient Uranium for U-Series dating within the wood almost certainly implies movement into the system after deposition, and thus it is doubtful that the Uranium system remained closed after this event (Duller et al., 2015). Instead, tt-OSL dates place the entire Package 1 from new excavations at Site C (correlated with the Mkamba Member) in a period from ~500-300 ka (Duller et al., 2015). This suggests that all Acheulean assemblages within the wider region are likely to fall into this period. The appearance of MSA technologies at Site C also takes place within the Mkamba member, and is bracketed by dates of 386 ± 94 ka and 455 ± 103 ka (below and above the archaeological horizon, respectively; Barham et al., 2015; Duller et al., 2015). Assuming the dates are correct at 1σ, this would suggest the ESA-MSA transition had already occurred by 352 ka, at the latest, and provides another minimum age constraint on Acheulean occupation. This 500-350 ka period means Acheulean hominins are likely to have been present repeatedly from MIS13-10.

The artefacts included in this study are taken from plan and lateral-view drawings included in the Kalambo Falls monograph (Clark, 2001). In order to increase the homogeneity of the assemblage, all artefacts derive from Horizon V of Site B, giving a total of 24 handaxes for investigation. Most artefacts are made on quartzite, but there is also rare presence of a "chert-like" material and silcrete (Clark, 2001). The assemblage is described as "Upper Acheulean", rather than "Final Acheulean", because it is not at the very end of the Acheulean succession at the site (Clark, 2001).

### 1.6. Kapthurin Formation

The Kapthurin Formation corresponds to the Pleistocene sedimentary sequence of the Tugen Hills, Kenya, to the west of Lake Baringo (Leakey et al., 1969; McBrearty and Tryon, 2005; Tryon et al., 2005). The formation covers an area of 150 km2 and has produced 70 known archaeological and palaeontological sites, but the region shares a common depositional succession (McBrearty and Tryon, 2005). The formation is split into five units, K1-K5 (from bottom to top), of which K1, K3, and K5 correspond to fluviolacustrine silts, sands, and gravels deriving from the drainage of the Tugen Hills basin (McBrearty and Tryon, 2005). These units were originally described as "torrent wash" by Leakey et al. (1969). Units

K1, K3, and K5 are separated by two major tephra members related to periods of intermittent vocanism: K2 (the Pumice Tuff Member) and K4 (the Bedded Tuff Member). A series of late Acheulean and early MSA assemblages have been found from the top of K3 and throughout K4 (McBrearty and Tryon, 2005; Tyron and McBrearty, 2002; Tryon et al., 2005).

The sample accessed for the present study derives from the initial surface collections and excavations of the 'Leakey Handaxe Site' (LHA, now known as GnJh-03; Leakey et al., 1969; McBrearty and Tryon, 2005). The sediments of the site derive from fluvial sediments of K3, above the Grey Tuff embedded within the unit (McBrearty and Tryon, 2005; Tryon et al., 2005). This tuff is 40Ar/39Ar dated to 509 ± 9 ka, providing a maximum age for the assemblage (Deino and McBrearty, 2002). Furthermore, an upper pumiceous layer of K4 has provided an age of 284 ± 12 ka, producing a minimum age for the assemblage (Deino and McBrearty, 2002). These age constraints make Acheulean occupation at the site possible anywhere between MIS13 and MIS8, although the stratigraphic relationship with K3, and the Grey Tuff more specifically, may imply an age in the earlier half of this timeframe.

The archaeological assemblage from the site is made on local phonolitic lava, and is notable for the presence of large Levallois cores to produce 10-20cm flakes (that were occasionally retouched into bifacial tools) and a Levallois and non-Levallois blade industry, alongside traditional Acheulean tool types (Leakey et al., 1969; McBrearty and Tryon, 2005; Tryon et al., 2005). 10 handaxe drawings or plates from Leakey et al.'s (1969) original publication were used to compile the data from the site.

1.7. Kharga Oasis

The Kharga Oasis palaeolandscape, near Kharga, Egypt, consists of a series of assemblages within spring-fed or overland flow tufa deposits, beginning in the Early Stone Age (ESA; Caton-Thompson, 1952; Kleindeinst et al., 2008). The present-day region is a desert with hyperaridity, but molluscan and floral remains indicate much wetter conditions during prehistoric occupations (Caton-Thompson, 1952). Stable isotope values and the presence of freshwater gastropods (*Melanoides tuberculate*) from the MIS5e even suggest that rainfall in the region was sufficient enough to sustain a small perennial lake during such periods (Smith et al., 2004). This line of evidence also points to limited climatic distinction between the different pluvial periods represented by the tufas (Smith et al., 2004), further increasing the likelihood that Acheulean hominins occupied the site during these wetter periods.

The most prominent Acheulean sites discovered by Caton-Thompson and Gardner are the KO10 Mound Springs Deposit, and at the base of the Refûf Pass sequence on the Kharga Escarpment (Caton-Thompson, 1952). At Refûf Pass, the "Upper Acheulean" artefacts of Caton-Thompson (1952) underly 'Tufa 1', for which U-Series dating gives a minimum age of ~400 ka (Kleindeinst et al., 2008). However, the artefacts included in the present study derive from KO10, with artefacts within both a comminuted clay and sands layer and a white sand layer (Caton-Thompson, 1952), which has unfortunately not been successfully relocated during modern survey and re-excavation projects (Kleindeinst et al., 2008: Table 1). Nonetheless, the assemblage of KO10 was initially described as "evolved" by Caton-Thompson (1952) due to the presence of handaxes that were converted into Levallois-like

cores, suggesting a character transitional between the ESA and the Middle Stone Age (MSA). As such, a tentative date of ~300-400 ka has been applied to the assemblage, based on the age of similar assemblages in the nearby Dakleh Oasis Palaeolandscape (Churcher et al., 1999; Kleindeinst et al., 2008). Combining this information with assignment to a pluvial, this may suggest hominin presence in MIS9, or perhaps MIS11.

A total of 23 handaxes were available for study from the Museum of Anthropology and Archaeology, Cambridge. All artefacts were made on chert, which is overwhelmingly the most common material at KO10 (Caton-Thompson, 1952).

**2. Levantine Sites**

2.1. Gesher Benot Ya'aqov

Gesher Benot Ya'aqov (hereafter GBY), located in the in the southern extension of the Hula Valley, Israel, preserves evidence for repeated human presence on the shores of Palaeo-lake Hula (Goren-Inbar, 2017; Goren-Inbar et al., 2018). The Acheulean artefacts of the site are located within the 34m of sediment comprising Benot Ya'aqov Formation, within which there are three main sedimentary inputs: lake margin beach facies (coquina, sand, and gravel sediments), shallow-water lacustrine facies (calcareous mud), and fluvial facies (river gravels). These sediments have been substantially tilted post-deposition by tectonic activity resulting from formation of the Hula Valley system and the Dead Sea Rift more generally (Feibel, 2004; Goren-Inbar, 2017).

The Acheulean artefacts are found within the lake margin sediments (Goren-Inbar, 2017; Goren-Inbar et al., 2018). The site may have acted as a repetitive home base for hominins over time, given there would have been access to year-round fresh water sources, lithic raw materials, and the abundant faunal and floral remains exploited extensively at the site, whereby the remains of the latter preserved at the site within the waterlogged sediments (Goren-Inbar et al., 2018). Hominins also habitually manipulated fire within the site (Alperson-Afil and Goren-Inbar, 2010, 2016). Deposition at the site documents five sedimentary cycles, lasting approximately 20,000 years each, related to fluctuations in the lake level, suggesting the >15 archaeological horizons throughout the sequence span a period of around 100,000 years (Goren-Inbar, 2017; Goren-Inbar et al., 2018). This is also supported by the progressive changes in geomagnetic field activity near the Brunhes-Matuyama boundary, documented within the Benot Ya'aqov Formation, which occurred over a period of 100-150,000 years at other sites (Goren-Inbar et al., 2000).

The entire sequence at GBY lies upon basalt flows identified in two drill cores that have been directly 40Ar/39Ar dated to ~1.3-1.1 Ma, providing a maximum age for the entire Benot Ya'aqov Formation (Goren-Inbar et al., 2012, cited by Goren-Inbar et al., 2018). Furthermore, the Brunhes-Matuyama reversal (~773 ka) takes place relatively near the top of the sequence which, given the chronostratigraphic knowledge for site formation time, implies the sequence spans ~0.8-0.7 Ma, with an estimated correlation to MIS20-18 (Goren-Inbar et al., 2000, 2018). The Early-Middle Pleistocene age is consistent with biostratigraphic considerations derived from the fauna (Goren-Inbar et al., 2000). This framework is further supported by a minimum age constraint of ~0.66 Ma obtained by 40Ar/39Ar dating of a

basalt flow interspersed with Benot Ya'aqov sediments below the archaeological horizons (Goren-Inbar, 2017; Goren-Inbar et al., 2012, cited by Goren-Inbar et al., 2018). This allows correlation to another (younger) Acheulean site in the region, the "North of Bridge Acheulian" (NBA) site, where the flow is dated to 658±15 ka (Sharon et al., 2010).

The archaeological assemblages are fairly homogenous throughout the ~100,000 years of GBY occupation, with non-linear differences in tool frequencies accounting for most variability (Goren-Inbar et al., 2018). The three raw materials exploited at the site (basalt, flint, and limestone) were used for distinct reduction sequences, resulting from differences in function (Goren-Inbar, 2017; Goren-Inbar et al., 2018). Basalt was used in percussion activities (anvils, percussors, and pitted stones), while its reduction accounts for almost all biface production at the site. This involved location of thick basalt slabs, their fragmentation, transformation into giant cores for production of large flakes, and final minimal shaping into bifaces (handaxes and cleavers; Goren-Inbar et al., 2018). This is typical of the Large Flake Acheulean (LFA; cf. Sharon, 2007, 2009). Flint working focused on reduction of small pebble-cores and flake-cores. Reduction sequences were not particularly structured, but 37.9% of products were retouched and standardisation was achieved by the removal of the bulb and striking platform to produce a flat surface that could be hafted (Alperson-Afil and Goren-Inbar, 2016; Goren-Inbar et al., 2018). Limestone was used for flaking or crushing percussors, and rarely flaked artefacts (Goren-Inbar, 2017; Goren-Inbar et al., 2018).

The artefacts included in the present study all derive from different sublayers of Unit II-6 (from oldest to youngest: Layers 4b, 4, and 1), the richest archaeological unit (Goren-Inbar et al., 2018). The base of this unit is located around 4m above the Brunhes-Matuyama reversal, and thus its age can be further refined to <773 ka (Goren-Inbar et al., 2000). The data were collected, and kindly provided, by Gadi Herzlinger and Naama Goren-Inbar, deriving from 3D models of the artefacts used elsewhere (e.g. Herzlinger et al., 2017; Herzlinger and Goren-Inbar, 2019, 2020). These three layers were selected for having >30 handaxes each, and all artefacts were made on dense Olivine basalt which, despite a minor flint and limestone component, dominate the assemblage (Goren-Inbar et al., 2018; Herzlinger and Goren-Inbar, 2019). 2D images were generated by G. Herzlinger and N. Goren-Inbar from the models imported into "Artifact GeoMorph Toolbox 3-D (AGMT3-D)" (Herzlinger and Grosman, 2018).

## 2.2. Nadaouiyeh Aïn Askar

The site of Nadaouiyeh Aïn Askar (henceforth Nadaouiyeh), is located near El Kowm, Syria, an area important for its cluster of natural springs that repeatedly attracted animals and humans over at least one million years (Le Tensorer et al., 2007). These water holes have formed as faults in the marly limestone and chalk bedrock have allowed water to be forced to the surface, creating a karstic system (Jagher, 2011; Le Tensorer et al., 2007). This process has resulted in the repeated erosion to the roof of the karstic system, leading to at least 7 collapse events and the formation of multiple dolines. These dolines have acted as natural sediment traps, preserving repeated evidence of human occupation of the dolines and their immediate surroundings (Jagher, 2011; Le Tensorer et al., 2007). This includes physical remains of a Homo erectus parietal in facies D (Schmid, 2015). However, collapse processes

have also destroyed some of the earliest sediments in the site, and have contributed to a complicated 32m stratigraphic succession through the tilting and lowering of sedimentary units (Jagher, 2011; Le Tensorer et al., 2007).

The sequence at Nadaouiyeh consists of six main depositional facies (from top-to-bottom: Nad-A to Nad-F; Jagher, 2011, 2016), with sedimentary input was distinct between periods of a high-water table and those of a low-water table. When water levels were high, the dolines filled with water, forming spring-fed ponds in which limnic sediments were deposited in a laminar manner (Jagher, 2011; Le Tensorer et al., 2007). There was also a littoral component to deposition around the outside of these ponds, and a minor travertine contribution within the pond deposits from the precipitation of calcium carbonate in warm, alkaline water (Le Tensorer et al., 2007). Dry periods greatly limited the amount of standing water available in the depressions, and thus deposition is overwhelmingly related to surface runoff into the dolines from spring output, exacerbated by the lack of surrounding vegetation around the spring in dry periods (Jagher, 2011; Le Tensorer et al., 2007).

Several attempts at radiometric dating of the Nadaouiyeh have failed, with only limited chronostratigraphic and palaeontological considerations available to produce an age model for the site (Jagher, 2011). This has been made even more difficult by the presence of numerous hiatus events of unknown duration between units. In addition, palynological data suggest an open steppe environment throughout the sequence (Renault-Miskovsky, 1998, cited by Jagher, 2011), and thus cannot be used as a palaeoclimatic indicator as the local subsoil is poor at holding water, preventing a wide spread of vegetation in wet periods (Le Tensorer et al., 2007). There are also few clear palaeontological indications of climatic change throughout the sequence (Reynaud Savioz, 2011).

The clearest chronological indicator in the sequence is a massive periglacial solifluction deposit in level c.7, at the base of Nad-B (Jagher, 2011). This deposit is highly likely to be correlated with the severe glacial period MIS12 (Jagher, 2011). As such, Nad-F to Nad-C are considered to date to MIS12 or earlier, with Nad-B and Nad-A post-MIS12. The post-MIS12 character of Nad-B and Nad-A is reinforced by the erosive contact and subsequent depositional hiatus resulting from the solifluction flow (Jagher, 2011), suggesting there cannot have been an immediate return to deposition. Dating of these later units is also helped by correlation to other Levantine sites, and the rare presence of late Lower and early Middle Palaeolithic industries at the site, namely the Yabrudian and the Hummalian (Jagher, 2011; Le Tensorer et al., 2007). These industries both lie in between the Acheulean assemblages of Nad-B and Nad-A, suggesting the Nad-B handaxes are associated with terminal Acheulean (*sensu stricto*, with a predominance of handaxes over retouched flakes; see Jagher, 2011) dates elsewhere in the Levant, and Nad-A handaxes are penecontemporaneous with the onset of the Levallois technique in the Levantine Mousterian (Jagher, 2011, 2016; Le Tensorer et al., 2007; cf. Shea, 2013). This places Nad-B around MIS11 and Nad-A around MIS7 in the current age model for the site (Jagher, 2011; Reynaud Savioz, 2011).

The “Upper Acheulean” character of the earlier handaxe assemblages, particularly Nad-F and Nad-E, may be suggestive of dates from MIS13, alongside other assemblages of such refinement in the Levant (Jagher, 2016), there are few correspondences between biface

form and typological period in the region (Shea, 2013). Nonetheless, Nad-F at the very base of the sequence derives from a travertine deposit that is likely to correlate with a warm period (and a high water table, resulting in a pond; Jagher, 2011, 2016; Kalbe et al., 2016; Le Tensorer et al., 2007). Furthermore, while the hiatuses between units are of unknown duration, it is clearly that at least some sedimentary units were deposited relatively quickly. In the case of Nad-D, it is clear that the sediments span a period of no more than a few thousand years (Pümpin, 2003, cited by Jagher, 2011, 2016). This leaves the potential for the units corresponding to MIS12 or earlier to have been deposited in a condensed amount of time. The ostracods in these units also show a clear three-phase transitional pattern, all through a high-water table, indicating a gradual transition from a palustrine wetland into a spring-fed pond with increasing salinity, ultimately resulting in brackish conditions (Kalbe et al., 2016; Le Tensorer et al., 2007). This single transitional phase may further support the attribution of Nad-F to Nad-C to successive time periods throughout the evolution of the environment surrounding the spring. As such, the current age model places Nad-F to Nad-D in MIS13, with Nad-C tentatively suggested to extend into MIS12 (Reynaud Savioz, 2011). However, these dates are extremely cautious and should not be taken as absolute.

The archaeological assemblage at Nadaouiyeh is overwhelmingly represented by bifaces (N = 12,415) directly shaped into the raw material, with cores, flakes, and flake-tools extremely rare throughout the sequence (Jagher, 2011, 2016). A majority of these artefacts (9941) are classical handaxes, but there is also a sizeable proportion of “bifacial tools”, which retain a cutting-edge but are more ad-hoc and have fewer series of removals (Jagher, 2011, 2016). However, in a large majority of cases, these artefacts still meet the definition of handaxes utilised for the present study, and thus only a limited number of these specimens were excluded for analysis. A total of 2084 artefacts derive are from secure context, with most others deriving from the solifluction flow (Jagher, 2011).

Amongst those securely assigned to Nad-F to Nad-A, there is a somewhat-paradoxical overall decrease in standardisation and refinement over time (Jagher, 2011, 2016). This is particularly clear when looking at the oldest handaxes (Nad-F), for which the investment in reduction has been described as far exceeding a merely functional purpose (Le Tensorer, 2006). While the trend is not clear-cut, there is a clear distinction between Nad-F to Nad-C and Nad-B to Nad-A, as the latter two facies show an abandonment of standardisation, a considerable number of bifacial tools, the use of poorer-quality (more) local raw material, and the presence of cores and flake tools, the former of which were exploited only expediently and flakes were difficult to distinguish from those of biface reduction (Jagher, 2011, 2016).

The material from Nadaouiyeh Aïn Askar is stored at present at the Institute for Prehistory and Archaeological Science of the University of Basel, Switzerland. A total of 735 bifaces from the Nadaouiyeh sequence were accessed for the present investigation, making a representative sample of the entire assemblage. A sample of 30 selected from each unit in the sequence, for which caliper measurements were also extracted from the Supplementary Information of Jagher (2016), in order to validate data extracted from the photographs.

## 2.3. Tabun

Tabun, Israel, is a cave site on the western slope of Mount Carmel, located around 45m above sea level (McPherron, 2003; Ronen, 2017). The cave consists of three discrete chambers (Front, Intermediate, and Inner), from which only the inner chamber's ceiling is preserved, including an 8 m-wide 'chimney' (Ronen, 2017). The site has been excavated in three phases, firstly by D. Garrod (1929-1934), then by A. Jelinek (1967-1972), and finally by A. Ronen (1975-2002; e.g. Garrod and Bate, 1934; Jelinek, 1982a, 1982b; Ronen et al., 2000). Each excavator used different notation to describe the sedimentary succession but, despite excavation at different levels and depths, the units described by each author can be roughly correlated with each other (e.g. McPherron, 2003; Ronen, 2017; Shimelmitz, 2015, 2020). Here, I will use Garrod's initial description of units (G-B and 'chimney'; see Ronen, 2017) because the material accessed for the present study derives from her excavations, and thus her description of the sequence provides the most secure context for the assemblages. Descriptions of studies working according to the Jelinek system will be presented in terms of the Garrod sequence according to the stratigraphic correlations suggested in Ronen (2017: Table 24.1).

The Tabun sequence is comprised of ~25m of sediment, one of the longest and most complete in the Levant (Ronen, 2017). Deposition took place in three distinct phases. The lowermost ~10m of sediment reflects aeolian deposition of quartz sand from the seashore, and is followed by 2m of wind-borne silts and a final ~10m of alluvial clays (Ronen, 2017). The Lower Palaeolithic material all derives from the lowest phase of deposition, comprising Garrod's Units G, F, and E (Ronen, 2017). Unit G is not considered here because the industry contains no bifaces.

Dating of Tabun F and E has been attempted by TL on burnt flints, which are abundant in the sequence, as well as through ESR dating (e.g. Grün and Stringer, 2000; Laukhin et al., 2000; Mercier and Valladas, 2003; Mercier et al., 2000). A radiothermoluminescence (RTL) date of 610 ± 150 ka is available for Tabun F, attesting to its antiquity compared to the overlying Acheulo-Yabrudian, but this includes a very large error margin (Laukhin et al., 2000). Again, while not ideal, the Upper Acheulean character of the assemblage, and the rather gradual transition to the Acheulo-Yabrudian (Shimelmitz, 2015), would tend to support attribution to the later phases of this date, perhaps within the period of MIS13-11.

A single TL date on burnt flint from Jelinek's Unit XIV gave an age of 415 ± 27 ka (Mercier et al., 2000), and this unit is now correlated with Garrod's Unit Ed, suggesting deposition of this layer in MIS11 (Ronen, 2017: Table 24.1). No ESR dates are available for this level. The TL dates from Units Ec and Eb place these levels at 302±27 ka and 324±31 ka, respectively, and may be among the most reliable in the sequence due to the heavily carbonated nature of the sediments from which the flints derive (Mercier and Valladas, 2003; Mercier et al., 2000). Indeed, the TL date for Ec is in close agreement with the early and late uptake ESR ages from one tooth within the unit (Early Uptake: 262 ± 32 ka, Late Uptake: 330 ± 43 ka; Grün and Stringer, 2000; Mercier and Valladas, 2003; Rink et al., 2004). Another tooth presented as deriving from the unit provided outlying ages (Grün and Stringer, 2000), but this specimen was selected from museum collections and may not have derived from the same stratigraphic unit (Mercier and Valladas, 2003). This provenance-sourcing issue may also account for the discrepancy between the TL age for Eb (324±31 ka) and the ESR Ages (Early Uptake: 180 ± 32 ka, Late Uptake: 195 ± 37 ka; Grün and Stringer, 2000; Mercier and

Valladas, 2003). As such, it is taken, here, that the TL date for Eb is more reliable, especially when accounting for the enhanced carbonation of the sediments surrounding the TL-dated artefacts. Subunit Ea at the top of the unit is TL dated to 267±22 ka and 264 ± 28 ka (Mercier and Valladas, 2003), overlapping with the combined ESR/U-Series dates (208 +102/−44) for the same unit (Grün and Stringer, 2000).

At least 20% of handaxe from different levels at Tabun are made on flakes, although this is likely to be an underestimate, with a larger proportion made on cobbles (McPherron, 2003). The lowermost Unit F contains a true "Upper Acheulean" assemblage, with a large proportion of (relatively small; McPherron, 2003) bifaces amongst the lithic toolkit (Ronen, 2017). While selected sub-units of Unit E (split from Ed at the base to Ea at the top) can contain similarly large quantities of bifaces (Shimelmitz, 2015), the Acheulean assemblage of Unit F does not contain any "Yabrudian"-type racloirs/scrapers (elongated flakes with invasive Quina retouch; Ronen, 2017; Shimelmitz et al., 2014a). The presence of this tool type alongside bifaces in Unit E has led to its description as "Acheulo-Yabrudian", with substantial variability in the relative proportions of each between sub-units (McPherron, 2003; Shimelmitz, 2015; Ronen, 2017). Amongst the wider Acheulo-Yabrudian sites in the Levant, biface proportions tend to be similar to Acheulean assemblages in Europe (Jagher, 2011), suggesting the regional typological distinction does not warrant exclusion of these assemblages from the present investigation.

The Tabun sequence provides an opportunity to investigate changes in late Lower Palaeolithic behaviour at a single site. Interestingly, there is an inverse relationship between the proportion of Yabrudian scrapers in a Tabun Unit E assemblage and the proportion of bifaces, whereby the latter are more heavily worked when they make up a larger proportion of the assemblage (McPherron, 2003). This trend may be part of a wider trend in which true Acheulean layers, and more biface-focused facies of the Acheulo-Yabrudian, show greater recycling of tools and raw materials throughout their use-life (Shimelmitz, 2015). Nonetheless, this relationship between Yabrudian scrapers and bifaces may imply a change in landscape use towards the end of the Lower Palaeolithic sequence, which may also be supported by the potential appearance of controlled fire usage in Unit E (Shimelmitz et al., 2014b). Furthermore, while a majority of artefacts throughout the sequence are made on relatively local flint (mostly high quality; McPherron, 2003), there is also a transition away from secondary cobbles and towards primary outcrops towards the later phases of the Acheulo-Yabrudian, as hominins invested in predetermined flake reduction schemes (Shimelmitz et al., 2014a, 2020).

Samples were compiled for assemblages, all accessed from the Marshall et al. (2002) biface database, derived from three of Garrod's units/sub-units: Units F, Ed, and Eb. As mentioned above, Unit F corresponds to a true Acheulean occupation, while Units Ed and Eb contain some of the largest biface assemblages in the Tabun Acheulo-Yabrudian (McPherron, 2003), and thus had statistically-relevant samples available on the database. 25 handaxes were available from Tabun F, with 71 and 62 available for Ed and Eb, respectively.

## 3. European Sites

### 3.1. La Noira

The open-air site of La Noira (near Brinay, Centre Region, France) is located within the Middle Valley of the Cher River, a tributary of the Loire in its Middle Basin. The deposits of La Noira belong to the Les Fougères Formation, one of the 9 stepped alluvial deposits of the Cher river system incised into the limestone bedrock. These sedimentary units derive from successive fluvial incisions and aggradations resulting from the characteristic climatic cycles of the Early and Middle Pleistocene (Despriée et al., 2016; Moncel et al., 2013; Voinchet et al., 2010).

The Les Fougères Formation at La Noira consists of five distinct strata, preserving evidence of two distinct occupations (Despriée et al., 2011; Iovita et al., 2017; Moncel et al., 2013, 2016, 2020a). Archaeological evidence for the earlier occupation (known throughout as "La Noira Lower") is present within stratum a. Stratum a corresponds to two diamicton deposits (sub-strata a1 and a2), resulting from glacial solifluction (Duval et al., 2020; Moncel et al., 2020a). The assemblage is present within the lower sub-stratum a1, for which the sediment is comprised of millstone slabs (siliceous lacustrine limestone), quincyte, Jurassic cherts, granite, and quartz nodules in a clayey-gravel matrix (Despriée et al., 2016; Duval et al. 2020; Moncel et al., 2013). This acidic quartztose deposit was not conducive to organic preservation, but use-wear evidence reveals a diversity of activities, including butchery (terrestrial fauna as well as fish and possibly birds), plant-working and wood-working (Hardy et al., 2018). The site is thus interpreted as multi-functional (both workshop and domestic activities; Hardy et al., 2018).

While post-depositional cryoturbation has affected the relationship between the sediments and the archaeological horizon in the northern portion of the site (Moncel et al., 2013), no such disturbance exists towards the south of the excavation. Geological and taphonomic analyses in this area of the site confirm the in situ production of much of the lithic assemblage (Despriée et al., 2011, 2016; Moncel et al., 2013, 2016, 2020a). The deposition of stratum a1 occurred following the end of river incision (at the beginning of a glacial period), with hominin activity abandoned before the partial cryoturbation of the site in full glacial conditions. This suggests an early glacial hominin presence before pleniglacial onset (Despriée et al., 2011, 2016; Duval et al., 2020; García-Medrano et al., 2022; Moncel et al., 2013, 2016, 2020a). Electron Spin Resonance (ESR) on optically-bleached quartz from the overlaying stratum b (two sequences of sandy alluvium) has given a mean age of 655 ± 55 ka, providing a minimum age for the stratum a occupation (Moncel et al., 2013; Voinchet et al., 2010). This age framework has recently been supported by a combination of ESR and thermally-transferred Optically Stimulated Resonance (tt-OSL) dating on stratum a2, which provided a minimum age of 690 ± 52 ka for the assemblage in a1 (Duval et al., 2020). The sediments also show normal polarity, placing hominin presence at the site in the Brunhes Chron (<773 ka; Duval et al., 2020). Given the glacial nature of the stratum a deposits, and the lack of frost cusp marks on the artefacts (implying rapid cover by stratum a2 and a close temporal relationship; Moncel et al., 2020a), the Acheulean occupation at La Noira Lower can likely be assigned to early MIS16 (676-638 ka; Duval et al., 2020; Moncel et al., 2013, 2016, 2020a, 2021), and probably before 650 ka (García-Medrano et al., 2022).

The lithic assemblage (~90% of which is made on local millstone slabs) shows a separation of pathways towards the production of both small-medium and large (>10cm) flakes, by more

or less structured reduction schemes (most commonly partial bifacial; Moncel et al., 2013). Retouch is fairly common, making up 23.4% of the entire flake assemblage (mostly scraper retouch), but is more common on large flakes (Moncel et al., 2020a). Finally, there is a diversity of bifacial tools (up to 7.4% of the assemblage), including finely-made handaxes (Moncel et al., 2013, 2016, 2020a, 2021). 136 bifacial tools were photographed for the investigation from the MNHN collection-Paris, at the Institut de Paléontologie Humaine (IPH), Paris, France (Part of the Muséum National d'Histoire Naturelle; MNHN). The specimen numbers for the artefacts selected for the final sample are:

BFLN 0.A7 d.2 no 1, BFLN 0.C5 d.1 no 1, BFLN 0.E3 d.1 no 1 ,BFLN 2.W9 d.1 no 1, BFLN 2.Z2 d.5 no 1, BFLN 2.Z5 d.1 no 1, BFLN 2.Z7 d.1 no 16, BFLNIII Secteur G A, BFNIII 105, BFNIII 110, BFNIII 113, BFNIII 142, BFNIII 144, BFNIII 145, BFNIII 148, BFNIII 15, BFNIII 155, BFNIII 158, BFNIII 184, BFNIII 187, BFNIII 200, BFNIII 260, BFNIII 32, BFNIII 39, BFNIII 44, BFNIII 52, BFNIII 68, BFNIII 77, BFNIII 81, and BFNIII 87.

The second hominin occupation at La Noira (known throughout as "La Noira Upper") is located towards the top of stratum c, a colluvial rubble diamicton layer overlain by a silty soil (stratum d; García-Medrano et al., 2022; Iovita et al., 2017; Moncel et al., 2013, 2016, 2020a). Stratum c is formed on an erosive surface that truncated ice wedges, suggesting hominins were present in an interglacial (Iovita et al., 2017). The top of the stratum (above the lithic assemblage) is also ESR dated to 449 ± 45 ka (García-Medrano et al., 2022; Iovita et al., 2017; Voinchet et al., 2010), but this is suspected to be an underestimate as the erosive surface may have resulted in the reworking (and exposure to sunlight) of the dated sediments and quartz grains (Duval et al., 2020). As such, La Noira Upper may date to MIS13, but is most likely to fall in MIS11 (Duval et al., 2020; Moncel et al., 2021).

The archaeological assemblage of stratum c is cleary distinct from that present in stratum a, with the biface assemblage made on both local millstone and non-local Jurassic cherts and flints from 30-100 km away (Despriée et al., 2011; García-Medrano et al., 2022; Iovita et al., 2017; Moncel et al., 2021). Furthermore, removals appear to be more numerous and more invasive, with final edge retouch more frequent, bifacial volume better controlled, and shape less variable (García-Medrano et al., 2022; Iovita et al., 2017), with some Levallois production perhaps also evidenced (Moncel et al., 2021; García-Medrano et al., 2022). In general, cores are most frequently bifacial and partially discoidal, with even most of the unifacial cores displaying centripetal scars (Moncel et al., 2021). 75 bifaces from this assemblage were accessed from the MNHN collection-Paris at the IPH, MNHN, Paris, for study, 30 handaxes from which formed the final sample. The specimens selected for investigation are numbered:

BFNVI 17, BFNVI 20, BFNVI 29, BFNVI 30, BFNVI 31, BFNVI 32, BFNVI 42, BFNVIB 101, BFNVIB 103, BFNVIB 105, BFNVIB 106, BFNVIB 107, BFNVIB 109, BFNVIB 113, BFNVIB 117, BFNVIB 125, BFNVIB 133, BFNVIB 134, BFNVIB 178, BFNVIB 180, BFNVIB 21, BFNVIB 241, BFNVIB 26, BFNVIB 295, BFNVIB 306, BFNVIB 324, BFNVIB 44, BFNVIB 46, BFNVIC 19, and BFNVIC 44.

### 3.2. Moulin Quignon

Moulin Quignon (near Abbeville, northern France) in the Middle Somme Valley is a classical Acheulean sequence and one of the first to reveal bifaces (see Antoine et al., 2019; Moncel et al., 2022). The artefact-deriving deposits of the site consist of a series of poorly sorted (fluvial) heterometric gravels and sands, containing chalk blocks and flint nodules in a calcareous matrix, overlying one of the Somme's terraces incised into the Cretaceous chalk bedrock (Antoine et al., 2019). The Moulin Quignon sediments also clearly derive from glacial conditions, not least demonstrated by the presence of large rounded tertiary sandstone blocks at the base of the sequence. These blocks have been reworked by periglacial mudflows, implying major episodes of solifluction (Antoine et al., 2019).

The Moulin Quignon deposits can be correlated with Formation VII of the Somme sequence, given the artefacts derive from similar absolute altitudes to those from the well-dated nearby sequence at Carrière Carpentier (Antoine et al., 2016, 2019). This correlation with Carrière Carpentier is crucial for understanding the stratigraphy and age of the Moulin Quignon site. The Acheulean artefacts from Carpentier are securely dated to MIS14, based on their intimate relationship to an underlying interglacial "white marl complex" that was unquestionably deposited in MIS15 (Antoine et al., 2016, 2019; see Carrière Carpentier section below). While the artefact-bearing deposits at both Moulin Quignon and Carpentier derive from glacial periods, the former are fluvial sediments while the latter represent a heterogenous and poorly sorted slope movement and thus the sites do not share identical sedimentary histories (Antoine et al., 2016, 2019). However, in the immediate area, other Formation VII sites between Moulin Quignon and Carpentier (Léon 1, Léon 2, and Chemin de Fer) reveal a consistent sequence of (from top to bottom) undifferentiated slope deposits (correlated to the MIS14 artefact deposits at Carpentier), a white marl complex (MIS15), and poorly-sorted heterometric gravels (correlated to artefact deposits at Moulin Quignon; Antoine et al., 2019, see Antoine et al., 2019: Figure S5). The (previously commercially exploited) undifferentiated slope deposits are likely to have previously directly overlain the fluvial gravels at Moulin Quignon, with the marl absent from the site. These geochronological correlations imply the fluvial gravels and sands at Moulin Quignon are from a glacial period older than MIS15 as the sediments underly the marl at other local sites (Antoine et al., 2019).

The great age of the site is confirmed by ESR dating on quartz from the fluvial gravels and sands containing the bifaces, giving a mean age of 672 ± 54 ka (Antoine et al., 2019; Moncel et al., 2022). Taking this information with the geological work and stratigraphic correlations with other sites, the Moulin Quignon Acheulean artefacts can be confidently assigned to MIS16, approximately coeval with La Noira and Notarchirico.

The archaeological assemblages of the site are split between old (19th Century) and new excavations, but the overarching stratigraphic observations of both are similar (Antoine et al., 2019). The new excavations revealed a relatively small assemblage of 254 flint artefacts, including 5 bifaces (Antoine et al., 2019; Moncel et al., 2022). Flakes tend to be medium-large in size (40-80 mm long) from both expediently and exhaustively exploited cores, but retouch is uncommon. Despite the small number, the bifaces show a great degree of variability in shape, reduction method, and refinement (Antoine et al., 2019). Plan and lateral view images derived from 3D scans of all of these bifaces are shown in Figure S8 of Antoine et al. (2019), one of which was excluded from the study due to a broken tip. An

additional handaxe held at the MAA in Cambridge was added from the old excavations, making a total sample of 5.

### 3.3. Notarchirico

Notarchirico (Venosa Basin, Basilicata) is an open-air site in Southern Italy that preserves evidence of repeated hominin occupation in a riverine-lacustrine context (Moncel et al., 2019; Piperno, 1999). Occupations are inferred from the presence of 11 distinct "archaeosurfaces" (containing lithic artefacts in a pebble-bed) throughout the 7m deposit (Moncel et al., 2019, 2023; Piperno, 1999). The site (particularly the upper levels) has also preserved abundant faunal remains, mainly of elephant (*Elephas antiquus*), fallow deer (*Dama clactonia*), and bovids (*Bos primigenius* and *Bison schoetensacki*; Moncel et al., 2019, 2023; Pereira et al., 2015; Piperno, 1999). The hominin activity levels are thus often interpreted as butchery sites, especially for the "Elephant Butchery Area" near the top of the sequence (Piperno and Tagliacozzo, 2001), but the taphonomic evidence for a functional association between lithics and animal remains remains poor (Moncel et al., 2019). Nonetheless, the importance of Notarchirico in the early European record is underlined by the recovery of a hominin femoral diaphysis from the α level at the top of the sequence (Piperno et al., 1990). The specimen is currently assigned to *Homo heidelbergensis* (Moncel et al., 2019), but was initially described by its similarity to Asian Homo erectus and more primitive traits (di Cesnola and Mallegni, 1996, cited by Roksandic et al., 2018; Piperno et al., 1990).

As the site is located just 10 km south-east of the Monte Vulture volcano, the Notarchirico sequence represents volcanoclastic sediments deposited and reworked within an alluvial environment (Pereira et al., 2015). The site is split into three distinct sedimentary units (from 3 at the base to 1 at the top), with the oldest non-biface assemblage (archaeosurface G) and the oldest biface layer (archaeosurface F) towards the bottom and top of the unit 3, respectively (Pereira et al., 2015). Archaeosurfaces E, E1, D, C, and B are found within unit 2, and A, A1, α, and sub-α derive from unit 1 (Pereira et al., 2015).

In the wider Venosa basin, there is evidence of three distinct units associated with the eruptive activity of Monte Vulture, the oldest of which (the Fonte del Comune Formation) is overlain by the Piano Regio and Tufarelle Formations which correlate to the early Middle Pleistocene (Lefèvre et al., 2010). On the basis of geochemical composition, the Notarchirico Tephra Complex (direct tephra fallouts of unit 2, levels 2.1-2.5) can be correlated with lavas and pumices of the Rinero Subsynthems, associated with the Piano Regio Formation and directly Argon-Argon (40Ar/39Ar) dated to 655 ± 25 ka (Lefèvre et al., 2010). More specifically, the Notarchirico Tephra of level 2.4 specifically resembles the Rinero Subsynthems M8 marker tephra, 40Ar/39Ar dated to 654 ± 11 ka (Lefèvre et al., 2010). This provides a minimum age for the archaeosurface F assemblage. In addition, at the very top of the sequence (unit 1, level 1.6), a level of reworked volcanic sands, resulting from the accumulation of ash falls in water, is geochemically correlated with a tephrostratigraphic marker of the Tufarelle Formation (Pereira et al., 2015; Raynal et al., 1998), suggesting the entire sequence is of early Middle Pleistocene age.

The age framework suggested by tephrochronology is also supported by direct 40Ar/39Ar, ESR, and thermoluminescence (TL) dating of the Notarchirico sediments. Pereira et al. (2015) carried out 40Ar/39Ar dating of unit 3 (below archaeosurface F), unit 2 (levels 2.1, 2.2 and 2.6), and unit 1 (levels 1.3, 1.5, and 1.6), and bracketed the entire sequence between 670 ± 4 ka (unit 3) and 614 ± 4 ka (level 1.6). These dates follow the stratigraphic sequence at 1σ, and suggest the entire sequence was deposited during MIS16 (Pereira et al. 2015). This is consistent with micromammalian and palynological data which suggest cold and open conditions, typical of an Italian glacial period (Piperno, 1999; Sala, 1990, 1999). Furthermore, a date of 652 ± 4 ka for level 1.5 (reworked tephras in fine clays and calcareous beds) provides a further age constraint for hominin activity as the layer also lies above the highest archaeosurfaces (α and sub-α; Pereira et al., 2015), suggesting all archaeological materials from the appearance of the Acheulean can be placed within the ~20 kyr span from 670 ± 4 ka to 652 ± 4 ka. These dates are consistent with a single ESR date on quartz of 657 ± 31 ka for level 2.6 (Pereira et al., 2015), and a TL date on quartz of 640 ± 40 ka for level 2.1 (Pilleyere et al., 1999). Taken together, Notarchirico is likely to be very similar in age to La Noira Lower (both early MIS16), but also provides extremely high temporal resolution for repeated hominin visits to the site, separated by no more than a few thousand years. Recent excavations suggests there are deeper occupations, including with bifaces, that extend into MIS17, but these samples were not studied for the present investigation (Moncel et al., 2020b, 2023).

The core-and-flake assemblage is rather simple and heavily influenced by core morphology (moreso on limestone than chert), but retouch can heavily alter the shape of the blank (Moncel et al., 2019). The bifaces also show limited diversity and standardisation, but many specimens display a clear ability to expertly manage bifacial volume (Moncel et al., 2019; Santagata, 2016). This characteristic is strongly biased towards chert bifaces, rather than those of limestone or quartzite (Moncel et al., 2019). Nonetheless, only some of these deposits (F, D, B, A, and A1) contain bifaces, and they are always limited in number (Moncel et al., 2019). The difference between levels is likely to reflect differences in groups or functions at the site, as technological clusters do not match onto the chronological sequence (Rineau et al., 2022). The entire sequence produced 32 bifaces, 29 of which were available for study at the Museo archeologico nazionale di Venosa, Venosa, Italy (Moncel et al., 2019). Permission for study of the artefacts was granted by the Ministero per i Beni e le Attività Culturali e per il Turismo – Polo Museale Regionale della Basilicata. Of these 29, a total of 20 were deemed to sufficiently meet the handaxe definition without major damage to the tip or laterals. Despite the fact that they derive from different archaeosurfaces, these handaxes were grouped together to make a single assemblage, for a more statistically relevant sample size.

## 3.4. Brandon Fields and Maidscross Hill

The sites of Brandon Fields and Maidscross Hill, Suffolk, UK, are just 4 km apart and both belong to the Bytham River Valley: a watercourse that ran from the Midlands to the East Anglia before being destroyed by the advance of the Anglian Ice Sheet in MIS12 (Moncel and Ashton, 2018; Moncel et al., 2015; Voinchet et al., 2015). This event dictates that all archaeological evidence associated with Bytham River sediments are pre-Anglian in age (but see Gibbard et al., 2009). Biface assemblages derive from the lowest two terraces incised

into the chalk bedrock by downcutting during interglacial conditions before MIS12. The lowest terrace (Terrace 1), at an altitude of around 10m (Bridgland et al., 1995), is thus likely to be MIS13 in age (Moncel et al., 2015), with the previous terrace (Terrace 2) inferred to be one interglacial older (Davis et al., 2017; Moncel et al., 2015).

Flower (1869) initially reported that biface assemblages from both Brandon Fields and Maidscross Hill were recovered within gravels rich in quartz and quartzite. This is now known to be a marker for the gravels of the Bytham river (Moncel et al., 2015). Furthermore, Ashton and Lewis (2005) report that the chalk bedrock at Maidscross Hill lies at an altitude of 21m above ordinance datum (AOD), much higher than known for the first terrace, seemingly confirming the attribution of the site to Terrace 2. This is very similar to the value measured by Flower (1869) for Brandon Fields, supporting a similar context (Davis et al., 2017). Indeed, the stratigraphically-lowest of 2 ESR dates on quartz from the sands and gravel from Maidscross Hill gave a date of 631 ± 56 ka (Voinchet et al., 2015), confirming that deposition at the site began before MIS13, and thus that the terrace derives from an earlier interglacial.

Nonetheless, caution must be taken associating the deposits from which the handaxes derive and the cutting of the terrace as a second ESR date from overlying sands and gravels at Maidscross Hill gave a slightly younger date of 529 ± 55 ka (Voinchet et al., 2015). While these dates do overlap at 1σ, the error margins of the second date leave open the possibility for some of the (likely interglacial, assuming correlation with Warren Hill; Bridgland et al., 1995) gravels to have been deposited in MIS13. As we lack information on the depth of the artefacts within these deposits, it is possible that the assemblages from Brandon Fields and Maidscross Hill are made up from the remnants of multiple isotopic stages (i.e. MIS15 and 13).

From a techno-typological perspective, Moncel and Ashton (2018) and Moncel et al. (2015) suggest the Brandon Fields and Maidscross Hill biface assemblages can be split into two broad groupings (both made on local flint): rolled, crude handaxes, produced with a hard hammer, and fresh ovate and condiform handaxes, shaped and retouched with a soft hammer. The assemblages of Brandon Fields and Maidscross Hill are dominated by the first of these categories (Moncel et al., 2015).

Specimens for both sites are stored at the British Museum (with those from Maidscross Hill labelled after the nearby town of "Lakenheath"; Moncel et al., 2015). A selection of 65 and 72 handaxes were photographed from Brandon Fields and Maidscross Hill, respectively.

### 3.5. Carrière Carpentier

The site of Carrière Carpentier (near Abbeville, northern France) is located in the Middle Somme Valley, around 40m above the present-day maximal incision of the waterway (Antoine et al., 2016). As mentioned above for Moulin Quignon, the Somme forms a regular stepped terrace system (Antoine et al., 2016, 2019), whereby the relative height of the Carpentier sediments implies correlation to Formation VII of this terrace (Antoine et al., 2016). The site, itself, consists of fluvial deposits topped with sediments deriving from slope processes (i.e. hillwash and solifluction; Antoine et al., 2016). The fluvial sediments can be

subdivided into two units, the lower of which is comprised of three gravel deposits. As at Moulin Quignon, the lowermost gravels are poorly sorted and contain large chalk blocks and flint nodules that likely derive from a glacial period, but the formations at the two sites are not directly comparable because the overlying gravels of the same unit at Carrière Carpentier are substantially better sorted, and appear to be reworked by early interglacial fluvial activity (Antoine et al., 2016).

The remainder of the fluvial sequence comprises a series of calcareous sandy silts and oncolithic sands that form the “White Marl Complex” (Antoine et al., 2016, 2019). These fluvial sediments are overlain by a series of slope deposits defined colluvial sands and thick clayey gravel beds resulting from solifluction (Antoine et al., 2016). The bifaces derive from these glacial deposits, which are dated based on their relationship to the white marl.

The White Marl Complex sediments are rich in carbonates, especially in the oncolithic lenses, precipitated through the activity of cyanobacteria. This biological marker is a clear indication of a permanent, calm freshwater environment in the temperate conditions provided by interglacial periods (Antoine et al., 2016). Furthermore, such conditions are also supported by the presence of aquatic mollusc species and fish species that indicate shallow, slow-flowing, or standing water (Antoine et al., 2016). The micro- and macro-faunal remains also suggest that the terrestrial environment showed a mosaic of woodland and open environments, typical of the spread of woodland associated with early interglacial periods (Antoine et al., 2016). Taken together, the White Marl Complex undoubtedly derives from interglacial fluvial sedimentation. Biochronological markers suggest these sediments are most likely to derive from a late Cromerian (pre-MIS12) context (Antoine et al., 2016). This notion is strongly supported by ESR dating on quartz and combined ESR/U-Series dating on macrofaunal teeth, which give a mean age of 584 ± 48 ka (Antoine et al., 2016). This securely places the unit within the earlier phases of MIS15, and provides a minimum age for the bifaces in the overlying unit.

The boundary between the White Marl Complex and the slope deposits from which the bifaces were excavated is strongly erosive, which indicates a relatively sudden shift from a fluvial to a terrestrial deposition environment (Antoine et al., 2016). Therefore, the deposition of the slope sediments is likely to correspond to the glacial period immediately following the deposition of the marl, MIS14. This would place the abandonment of the bifaces either within early MIS14 itself, or at the end of MIS15, only to be incorporated into the glacial slope movement (Antoine et al., 2016). Either of these scenarios is plausible, but the assignment of the sediments to MIS14, and the absence of evidence for human occupation in the immediate vicinity during the White Marl Complex (Antoine et al., 2016), may make an early glacial presence more parsimonious.

The modern excavations revealed five finely-made bifaces on local flint nodules. These artefacts are held as part of the MNHN collection-Paris at the IPH, MNHN, Paris, all of which were used in the present study. These have the following specimen numbers:

CC, CC2012(1), CC2012(2), CC2013 G4 5 Sup, and CC2013 H4 3 Sup.

### 3.6. Warren Hill

As for Brandon Fields and Maidscross Hill, the quartz and quartzite-rich gravels of Warren Hill, Suffolk, UK, derive from deposition of the Bytham River, destroyed by Anglian glaciation (Moncel et al., 2015). However, in contrast to those sites, the chalk bedrock is found at an altitude of 10m AOD (Bridgland et al., 1995), assigning it to the lowest terrace of the Bytham River system (Terrace 1; Westaway, 2009, but see Lee et al., 2004), thought to date to MIS13 (Moncel et al. 2015). ESR dates for the Warren Hill gravels suggest ages of 544 ± 53 and 539 ± 38 ka (Voinchet et al. 2015), may suggest deposition either in MIS15 or MIS13 (as the gravels do not derive from glacial context; Bridgland et al., 1995). Thus, taken together, it is highly likely that the handaxes from Warren Hill derive from MIS13.

The number of handaxes recovered from the site is extremely large (Moncel et al., 2015), and photographs of 341 specimens, along with morphometric data, are accessible on the Marshall et al. (2002) biface database. However, the flint handaxes can be also split into the same two groups as Brandon Fields and Maidscross Hill, with a rolled, more crudely-worked, hard-hammer-only set of handaxes, and a number of fresh, finely worked specimens, that involve the use of a hard-hammer (Bridgland and White, 2015; Moncel et al., 2015). Roe (1968) suggested that distinctions in morphometric measurements between the two forms warranted a technological separation into two distinct assemblages at Warren Hill, with the more worn falling into Group V with other 'crude' handaxe assemblages, and the fresher specimens contributing to Group VII (well-made ovates). As such, two samples were selected for the present investigation. A "worn" assemblage was compiled by selecting only those whose condition was described as "Rolled" or "Abraded", alongside a patination described as "Extreme Patination" or "Patinated", on the Marshall et al. (2002) database. 30 specimens were randomly selected from this subset to complete the assemblage. In contrast, a "fresh" assemblage was established by random selection of 30 specimens described as "Fresh" or "Lightly Abraded", with little or no patination.

### 3.7. High Lodge

High Lodge, Suffolk, UK, is the final site sampled from the Bytham River Valley, and the only one of the four excavated rather than recovered from unrelated activities (Ashton et al., 1992). The sequence at High Lodge consists of a series of fluvial sands and gravels, overbank clayey-silts, and glaciofluvial sands deposited during MIS12 (Brumm and McLaren, 2011; Moncel and Ashton, 2018; Moncel et al., 2015; Lewis, 1992; Rose, 1992). This attests to the pre-Anglian credentials known from the other Bytham River sites (but see Gibbard et al., 2009; West et al., 2014). Furthermore, an age older than MIS12 is suggested by the presence of part of a tooth from the rhino *Stephanorhinus hundsheimensis*, which went extinct in MIS12 (Stuart, 1992).

The overbank sediments of Beds B and C have produced a rich in situ core-and-flake assemblage without handaxes, characterised by extremely invasive scraper retouch (Ashton, 1992; Moncel et al., 2015). These sediments have undergone subglacial deformation, resulting in them overlying glacial till, but are still covered by subsequent glaciofluvial sand (Lewis, 1992; Moncel et al., 2015). This implies a close-temporal relationship to the Anglian, suggesting a late MIS13 age. Indeed, this is also consistent with palynological and coleopteran data, which indicates a cool temperate environment (Ashton and Lewis, 2012;

Coope, 1992; Hunt, 1992). The flint handaxe assemblage at High Lodge, however, is in secondary position, and derives from the base of the MIS12 glaciofluvial sands (Moncel and Ashton, 2018; Moncel et al., 2015). This implies that the handaxes derive from a different part of the floodplain during MIS13, potentially coeval with the core-and-flake assemblage (Moncel and Ashton, 2018; Moncel et al., 2015).

The handaxe assemblage included in the investigation is described as similar to the fresh handaxes from Warren Hill (Moncel et al., 2015), also assigned to Roe's Group VII (Bridgland and White, 2015; Roe, 1968; White, 2015). 66 handaxes held at the British Museum were photographed from High Lodge for the present study.

### 3.8. Boxgrove

The deposits of Boxgrove (Eartham Quarry) on the West Sussex Coastal Plain, UK, are situated on the highest (~40m AOD) of a series of platforms cut into the Cretaceous chalk bedrock by tidal activity during periods of high sea levels, likely interglacials (Roberts and Parfitt, 1999). These sediments have been protected from coastal erosion by tectonic upflit since the early Middle Pleistocene (Roberts and Parfitt, 1999). The stratigraphy of the site complex can be split into two formations: the Slindon Formation (Units 2-5a) and the Eartham Formation (Units 5b-11), with archaeological and faunal remains found throughout both units (Roberts and Parfitt, 1999). The Slindon Formation was formed by mostly marine modes of deposition, whereas the Eartham Formation consists entirely of terrestrial slope runoff, slope deposits, and cliff collapse, with sediments deriving from the north of the chalk cliff that forms the back of the sedimentary sequence (Roberts and Parfitt, 1999).

While both formations are of archaeological importance, Units 4b and 4c of the Slindon Formation preserve the vast majority of in situ or minimally-disturbed deposits known from Boxgrove, and provide the best chronological resolution. The formation is divided into the Slindon Gravel Member (Unit 2), the Slindon Sands Member (Unit 3), the Slindon Silt Member (Unit 4), and an organic bed (Unit 5a). Units 2 and 3 (cyclical littoral deposits) preserve only limited archaeological remains and Unit 5a is sterile of lithic artefacts (Roberts and Partfit, 1999). The deposits of Units 4a-b reflect a transition to a lagoonal environment, facilitating twice-daily deposition of silty sediments in a low-energy mudflat environment, and rapid covering of the archaeological remains in Unit 4b (Roberts and Parfitt, 1999). There is no sedimentological evidence of vegetational activity in this layer (Macphail, 1999), but there is some evidence of root corrosion on bones which may suggest it was at-least occasionally present (Roberts and Parfitt, 1999). In contrast, the reduction in sea level evidenced by Unit 4c exposed the mudflats to subaerial weathering and drying-out, allowing a productive soil horizon to form. This is likely to have been present for 20-100 years, implying all archaeological material from the unit derives from this period (Roberts and Parfitt, 1999).

Chronometric dating of Boxgrove has proven extremely difficult, and has resulted in huge error margins and inaccuracies (Roberts and Parfitt, 1999). Nonetheless, the site can be correlated to other English sites on the basis of the vertebrate fauna, and strongly indicate a pre-MIS12 age for the site complex. For example, the shrews *Sorex runtomensis* and *Sorex savini*, the vole *Pliomys episcopalis*, the cave bear *Ursus deningeri*, the rhinoceros

*Stephanorhinus hundsheimensis*, and the giant deer *Megaloceros dawkinsi* and *Megaloceros verticornis* all went extinct in MIS12, and are all present within the Boxgrove Fauna (Roberts and Parfitt, 1999). This rhinoceros species is also known from other MIS13 handaxe sites from the UK, including High Lodge and Happisburgh-1 (Moncel et al., 2015; Stuart, 1992). Faunal climatic indicators also suggest that the deposition of the main archaeological Units (4b and 4c) occurred after the interglacial optimum, suggesting occupation dates to the latter stages of MIS13 (Roberts and Parfitt, 1999). Indeed, human presence continues into the glacial slope sediments of the Eartham Formation, suggesting hominin presence into MIS12 (Roberts and Parfitt, 1999).

Boxgrove is known for its extremely refined ovate handaxe assemblage made on local flint nodules from the base of the cliff, frequently involving a tranchet blow to the tip to produce an extremely sharp cutting edge (Roberts and Parfitt, 1999). These handaxes are made by distinct "roughing-out" (hard hammer or cortical flint hammer), thinning (soft hammer), and finishing (soft hammer) phases (Moncel et al., 2015; Roberts and Parfitt, 1999). There is very little archaeological change throughout the sequence, even in the face of the transition to a glacial period (Roberts and Parfitt, 1999). The shape of these handaxes is extremely homogenous (e.g. Iovita and McPherron, 2011; Iovita et al., 2017), and cores and retouched flakes are extremely rare (Roberts and Parfitt, 1999).

An assemblage of 30 handaxes was randomly selected from the 183 documented in the Marshall et al. (2002) biface database. Boxgrove sub-site was only recorded for 3 of these specimens, which derive from Q1/B. Nonetheless, all the handaxes were excavated in 1995-1996, in the same field seasons as the Q1/B specimens, and a large majority (32/33) of artefacts in the entire Boxgrove database sample also derive from this site. This makes it likely that most of the sample taken for the present study were also from this site. Furthermore, while stratum was not recorded, an overwhelming majority of bifaces from this site derive from spring-fed waterhole sediments coeval with the deposition of the Unit 4c soil horizon (García-Medrano et al., 2019; Pope and Roberts, 2005; Roberts and Parfitt, 1999). All the handaxes in the sample of 30 are also described as either "Fresh" or "Lightly Abraded", making it unlikely that they derive from the rolled handaxe assemblages of Units 2-3 or many of the glacial units (Roberts and Parfitt, 1999). This would place most of the handaxes from the same <100 year period in mid-late MIS13, but other rare specimens may be included from different periods.

### 3.9. Saint-Pierre-Lès-Elbeuf

The 18 m-thick loessic sequence of Saint-Pierre-Lès-Elbeuf, Seine Valley, France, consists of thick glacial loess units, interspersed between four well-developed interglacial palaeosols, notated Elbeuf I-IV from youngest to oldest (Cliquet et al., 2009; Leroyer and Cliquet, 2010). The sequence lies upon a terrace incised by the confluence of the Seine with a tributary, the River Oison (Leroyer and Cliquet, 2010). Elbeuf I is firmly correlated with MIS5, with the most parsimonious correlation for the other palaeosols corresponding to successive preceding interglacials, up to MIS11 for Elbeuf IV (Leroyer and Cliquet, 2010). This is also supported by the presence of a limestone tufa deposit overlying Elbeuf IV (vertebrate and mammalian fauna is clearly interglacial), which is likely correlated with MIS11 (Cliquet et al., 2009; Leroyer and Cliquet, 2010; Moncel et al., 2015), similar to the site of La Celle further

up the Seine sequence (Limondin-Lozouet et al., 2010). The loess overlying this tufa is likely to derive from MIS10 (Leroyer and Cliquet, 2010). ESR and combined ESR/U-Series dates are consistent with this MIS11-10 framework (Voinchet et al., 2015).

The site has produced a large number of handaxes resulting from quarry activity since the late 19th Century, with limited stratigraphic information (Leroyer and Cliquet, 2010). However, modern excavations at the site have revealed the presence of at least two main Acheulean occupations at the site, between Elbeuf III and Elbeuf IV (Moncel et al., 2015). The lower assemblage is located below the tufa, consisting of large, pointed, and elongated bifaces that tend to have well-worked tips. In contrast, the other assemblage shows substantial variation in size and form, often showing thick butts and twisted or plano-convex sections, and is located above (or within) the tufa (Leroyer and Cliquet, 2010; Moncel et al., 2015). Both assemblages are made on local flint nodules from the river gravels (Leroyer and Cliquet, 2010; Moncel et al., 2015). For the present investigation, 50 handaxes from the site were accessed from the MNHN collection-Paris at the Musée de l'Homme, MNHN, Paris, likely from the old collections at the site. The 30 randomly-selected handaxes have the specimen numbers:

D.38.23.23.965, D.38.23.31098, D.38.23.31099, D.38.23.31122, D.38.23.31456, D.38.23.31457, D.38.23.7455, D.38.23.7456, D.38.23.7457, D.38.23.7458, D.38.23.7460, D.38.23.7464, D.38.23.7468, D.38.23.7470, D.38.23.7475, D.38.23.7483, D.38.23.7485, D.38.23.7487, D.38.23.7491, D.38.23.7494, D.38.23.7497, D.38.23.7499, D.38.23.7501, D.38.23.7505, D.38.23.7506, D.38.23.7507, D.38.23.7509, D.38.23.7511, D.38.23.7522, and D.38.23.7534.

Leroyer and Cliquet's (2010) study of the old collections from the site suggest they derive from three different techno-typological assemblages: one from the Mousterian, another from the Micoquian, and finally one from the Acheulean. The authors suggest the Mousterian handaxes are small ovate and cordate handaxes, all showing a deep-white patination, which is indeed present in a small number of the final sample. However, these are not, themselves, characteristics which can be used to remove handaxes from the sample as they form a part of the Acheulean handaxe variation from the site. Indeed, this patination is also characteristic of an in situ assemblage from within the Saint-Pierre-Lès-Elbeuf tufa (Leroyer and Cliquet, 2010), so removing handaxes based on this trait could potentially bias the sample. There may also be one Micoquian handaxe (extremely elongated with a pointed tip), but not that would extend outside of normal Acheulean variation. This may mean that the sample contains artefacts which do not derive from the two main Acheulean contexts that are associated with the MIS11-10 interglacial cycle.

Nonetheless, these potentially-outlying handaxes make up a relatively small proportion of the sample (~6/30), and Leroyer and Cliquet (2010) suggest the remaining specimens may be assigned to a collection from Chedeville (1896, cited by Leroyer and Cliquet, 2010), said to derive from below the Elbeuf IV palaeosol. This would give a minimum age of MIS11. Alternatively, many of the specimens may derive from units equivalent to the modern excavations, from different parts of the site. Indeed, the elongated and pointed handaxes that may derive from the Micoquian are also characteristic of the lower assemblage from the modern excavations, while the variety of shapes associated with the upper assemblage

(including particularly thick specimens) are also accounted for in the assemblage (Moncel et al., 2015). Thus, while it is extremely tentative, the majority of the handaxe sample from Saint-Pierre-Lès-Elbeuf is tentatively correlated with the excavated assemblages from MIS11.

### 3.10. Saint-Acheul

The gravel pits of Saint-Acheul, near Amiens, northern France, are famous for its large collection of handaxes that helped to establish the antiquity of lithic technology, giving its name to the Acheulean (de Mortillet, 1872) and helping to establish Palaeolithic archaeology more broadly (Commont, 1909, 1911; Prestwich, 1860; Rigollot, 1854). The sediments meet the chalk bedrock at 44m AOD which correlates them with the Garenne Formation, the 5th alluvial terrace of the Middle Somme Valley (Antoine and Limondin-Lozouet, 2004).

The sedimentary succession at Saint-Acheul is very similar to other sites in the Somme Valley, as deposition is split into a fluvial phase (Units 1-3), from which the archaeological material derives, and a terrestrial phase (Units 4-14), consisting of loess, colluvial runoff, palaeosols, and solifluction gravel beds (Antoine and Limondin-Lozouet, 2004). The fluvial accumulation begins with poorly sorted heterometric flint gravels that correspond to deposition in a glacial period (Antoine and Limondin-Lozouet, 2004), much like at Moulin Quignon (Antoine et al., 2019). This is succeeded by a calcareous sandy loam (Unit 2) and a calcareous tufa deposit (Unit 3). These units are characterised by a diverse interglacial malacofaunal assemblage (Limondin-Lozouet and Antoine, 2006), with the small oncoliths of Unit 3 also confirming the temperate climate (Antoine and Limondin-Lozouet, 2004), as in the calcareous marl at Carrière Carpentier (Antoine et al., 2016).

Within the great diversity of molluscs recovered from the site, those from open grassland environments dominate amongst terrestrial species, while aquatic species suggest slow-moving water (Antoine and Limondin-Lozouet, 2004). There is also a large cohort of shade-demanding species, indicating an important woodland component, which increase throughout Unit 2 and peak in the tufa, from the top of which the handaxes derive. The tufa also contains a large number of thermophilic species, which, taken together with the increasing woodland cover, suggests Unit 3 corresponds to an interglacial optimum. The presence of *Retinella skertchlyi* is also informative, as it is mostly found in other western European sites dating to MIS11 (Antoine and Limondin-Lozouet, 2004; Limondin-Lozouet and Antoine, 2006). This age is supported by an ESR date from underling Unit 2 of 403 ± 73 ka (Bahain et al., 2001). This firm assignment to an interglacial peak of MIS11 is likely to correlate with MIS11c.

Saint-Acheul has produced a large number of flint handaxes from different artefact collection episodes and excavations, now stored at multiple different museums in France and the UK. For this investigation, 42 handaxes held at the Museum of Anthropology and Archaeology, UK, were photographed.

### 3.11. Elveden

The site of Elveden, Suffolk, is located in the same geographically-restricted region (Breckland) as the Bytham River Valley sites (Ashton et al., 2005). The sediments of the site fill a solution depression in the Chalk bedrock, which is immediately overlain by a poorly sorted chalk and flint diamicton, with a silt and clay matrix, that constitutes Anglian till (Ashton et al., 2005). This necessitates that occupation of the site was younger than MIS12, and thus derives from after the glacial destruction of the Bytham drainage system.

The subsequent sediments of Elveden consist of a grey silty-clay deposited under low-energy fluvial or lacustrine conditions, with an alluvial lag gravel and black clay (for which micromorphological evidence suggests it is the remnants of a palaeosol) on the margins of the solution feature (Ashton et al., 2005). These sediments are topped by fine-grained colluvial brickearth deposits before a final layer of Late Pleistocene aeolian coversands (Ashton et al., 2005). The mollusc, ostracod, and fish fauna, together, suggest a permanent, wide, and well-oxygenated water body, with slow-moving to still water, most likely a river, in temperate conditions (Ashton et al., 2005). The pollen paints the picture of an open and cool interglacial environment, but does not correspond to an interglacial peak due to the lack of arboreal species. Nonetheless, these do progressively increase throughout the fluvial/lacustrine, alluvial, and colluvial sequence, suggesting an early interglacial position with improving climatic conditions (Ashton et al., 2005).

With regards to the age of the site, the lithic assemblage derives from the lag gravel, palaeosol, and lower brickearth deposits, for which the underlying Anglian till requires occupation to have taken place in an interglacial after MIS12. The pollen profile of the site correlates with Hoxnian (MIS11) pollen zones Ho I and Ho IIa, which confirm the early interglacial character and suggest an age of early MIS11c (Ashton et al., 2005). This is supported by Amino Acid Racemisation (AAR) on mollusc shells, from which the D/L isomer ratios compare well with other MIS11 sites in the UK (Ashton et al., 2005).

The lithic assemblage of Elveden is famous for its large quantities of flint ovate handaxes recovered since the later 19th Century (Ashton et al., 2005). A large proportion of these (36-40%) show the characteristic S-twist in profile view characteristic of ‘twisted ovates’ that are common amongst East Anglian sites of MIS11c (Moncel et al., 2015; White et al., 2019). A total of 35 handaxes held at the Museum of Anthropology and Archaeology, Cambridge, UK, were accessed for study.

### 3.12. Swanscombe Middle Gravels

Swanscombe, Kent, UK, has been excavated at a number of productive Lower Palaeolithic sites since the early 20th Century (Conway et al., 1996; Smith and Dewey, 1913; Wenban-Smith, 2013; Wenban-Smith and Bridgland, 2001). The most famous site (and complete geological sequence) is at Barnfield Pit, from which the Swanscombe hominin skull derives (Ovey, 1964). The depositional sequence at Swanscombe is split into Phases I-III, the first two of which are fluvial and the third represents both slope and alluvial sediments (Davis and Ashton, 2019; White et al., 2013, 2019). Within the fluvial sediments, Phase I consists of the lower gravels and loams, while Phase 2 comprises the Middle Gravels (White et al., 2019; Davis and Ashton, 2019). The initial Phase III deposits (Upper Sands and Gravels) show evidence of cryoturbation and ice-wedges, suggesting a cold climate, in stark contrast to the

overlying loam which represents an interglacial estuarine deposit (Conway, 1996; White et al., 2019). The entire sequence rests on the Boyn Hill Terrace of the Lower Thames (Davis and Ashton, 2019; White et al., 2019).

The 'Rhenish' molluscan fauna of the Middle Gravels, which appear elsewhere in the Thames Valley in Hoxnian (MIS11) Ho-IIIa pollen zone place the Phase II sediments within mid-late MIS11c and, by extension, the Phase I deposits to early-mid MIS11c (White et al., 2013, 2019). Despite following a cold period which might be expected to represent MIS10, Schreve (2001) points out that the Swanscombe Unit III fauna do not correlate with MIS9. This may imply an alternative correlation of the cold period to the climatic downturn of MIS11b, with the subsequent warm period representing the climatic recovery of MIS11a (Schreve, 2001; White et al., 2013, 2019).

The three phases of Swanscombe deposition also correlate with three different archaeological assemblages, all made on local flint, with Phase I showing flakes and flake tools (but no handaxes) typical of the Clactonian (Moncel et al., 2015; White et al., 2019). Both Phase II and III show handaxe assemblages, but the former is well-known for its small sub-triangular or condiform pieces (mostly from the Upper Middle Gravels) in contrast to the frequently-twisted ovates of Phase III (Moncel et al., 2015; White et al., 2019). The present study deliberately targeted handaxes only from the Middle Gravels because of their larger sample size and because no other assemblage included in the study belongs to Roe's (1968) pointed Group II handaxes (see Bridgland and White, 2015; White, 2015). It was felt necessary to include in order to capture as much variation in European handaxe shape as possible.

47 handaxes from Swanscombe, stored at the British Museum, London, were accessed for the investigation. All of these artefacts belong to a collection donated by the Wellcome Institute for the History of Medicine in 1982, which, while no specific pit is described, are almost certain to derive from Barnfield Pit as other sites were excavated more recently than this date (e.g. Wenban-Smith, 2013; Wenban-Smith and Bridgland, 2001). Only handaxes from this collection were investigated in an attempt to minimise the chances of the specimens deriving from multiple sources. Nonetheless, it is unknown if they derive from the extensive excavations of the Upper Middle Gravels (from which the Swanscombe skull was excavated) carried out by Wymer in the 1950s (Moncel et al., 2015; Ovey, 1964) or an earlier collection, but the very limited number of handaxes found in the Lower Middle Gravels during more recent excavations (Conway, 1996) suggest they are likely to derive specifically from the Upper Middle Gravels regardless. Furthermore, the descriptions of the handaxe shapes (especially sub-triangular or pointed), as well as the described states of abrasion expected for an assemblage in a secondary position (Moncel et al., 2015), underline confidence that these specimens derive from the Swanscombe Middle Gravels.

### 3.13. Bowman's Lodge

Bowman's Lodge is a former gravel pit near Dartford, Kent, UK, comprising a sequence of river gravels (Dartford Heath Gravels) overlain by the silts and clays of the Wansunt Loam (White et al., 2019). Faunal remains from the Dartford Heath Gravels, including the extinct elephant *Palaeoxodon antiquus*, clearly point to interglacial conditions during the deposition

of the gravels (Chandler and Leach, 1912; Leach, 1913, cited by White et al., 2019). Furthermore, Newton (1895, cited by White et al., 2019) reported a series of molluscan fauna from the Dartford Heath Gravels at Dartford Brent nearby, including species characteristic of the ‘Rhenish’ fauna that appear in the Thames Valley in MIS11c, and that were used to date the Middle Gravels at Swanscombe (White et al., 2013, 2019). The overlying loam sediments have a maximum vertical extension of 42m AOD, which is extremely similar to the MIS11a Swanscombe Loam at the Swan Valley Community School Site (Wenban-Smith and Bridgland, 2001; White et al., 2019). This likely places Bowman’s Lodge onto the Boyn Hill Terrace alongside Swanscombe (Wenban-Smith and Bridgland, 2001; White et al., 2019). Together, this evidence suggests a very similar depositional history to Swanscombe, likely covering both warm periods of MIS11.

The known flint handaxes from Bowman’s Lodge derive from the top of the Dartford Heath Gravels and into the Wansunt Loam, likely placing them MIS11a (Tester, 1951, 1975, cited by White et al., 2019). It is characterised by a large proportion of twisted ovate forms (31-33%), that may be shared by many Thames valley sites in MIS11a (White et al., 2019). 29 specimens were available for the investigation from the Marshall et al. (2002). One artefact defined by Marshall et al. (2002) as a cleaver was conservatively excluded, even though the transverse edge was formed by a tranchet blow that would usually relate to handaxe reduction. This left a final sample of 28 artefacts.

### 3.14. Broom Pits

The three gravel pits of the Axe River at Broom, Dorset, have yielded >1,800 handaxes unearthed during quarry activity (Hosfield and Chambers, 2008). The terrace upon which the fluvial deposits lie was cut into the Foxmould-Whitecliff Chert Member of the Upper Greensand Formation (Hosfield and Chambers, 2008). Recent geological work (cf. Green, 1988) refined previous interpretations of the sedimentary sequence, describing it in terms of three distinct gravel units: the Lower Gravels, the Middle Beds, and the Upper Gravels (see Hosfield and Chambers, 2008; Green and Hosfield, 2013). The well-stratified Lower Gravels have much greater flint component (rather than chert) to its lithology, and they are separated from the Middle Beds by a sharp erosive boundary (Hosfield and Chambers, 2008). The Middle Beds have a finer sedimentary input, and palynological data suggests it corresponds to a temperate phase in-between colder periods (Hosfield and Chambers, 2004, 2008; Shakesby and Stevens, 1984). Nonetheless, lenses of sand and loam in the Upper Gravels may indicate at-least brief ameliorations in climate during the cold periods (Hosfield and Chambers, 2008; Toms et al., 2005).

Dating of Broom has proven more difficult than many other sites in the British Acheulean because it does not belong to a multi-terrace fluvial system that allows correlation between sites (Hosfield and Chambers, 2008; Green and Hosfield, 2013). Nonetheless, Bayesian modelling of OSL dates from the Middle Beds and Upper Gravels suggests respective age ranges of 324-284 ka and 292-205 ka for these units (Toms et al,. 2005). These dates correspond to Marine Isotopic Stages 9-8 and 8-7, with a plausible restriction of MIS9-8 imposed by the overall cold-period deposition of the Upper Gravels. The artefacts from Broom derive from throughout the fluvial sequence, and are likely to be in secondary context, given many are rolled and abraded (Hosfield and Chambers, 2008).

The Broom bifaces (mostly handaxes but also cleavers) are characterised by a large amount of morphological diversity, both in raw shape and symmetry (Hosfield and Chambers, 2008). They are also overwhelmingly (94.3%) manufactured on local Greensand chert, rather than flint (5.6%) which is a lot sparser in the local area (Hosfield and Chambers, 2008). 253 bifaces were available for study through the Marshall et al. (2002) database, for which the proportion of flint specimens (4.35%) closely matched the value suggested by Hosfield and Chambers (2008). 30 handaxes were randomly selected from this sample for the present study, with 29 being made on chert and 1 on flint.

3.15. Cuxton

The site of Cuxton in the Medway River Valley, Kent, revealed a large biface assemblage from several pits in which the sediments contact the chalk bedrock at heights of 14-17m AOD (Wenban-Smith, 2004, 2006). These fluvial deposits consist of a series of sands and gravels of restricted-depth, representing a small erosional remnant of Medway terrace deposits (Bridgland and White, 2015; Wenban-Smith, 2004). These limited deposits, alongside the lack of a clear terrace sequence in the valley, have made chronostratigraphic correlation extremely difficult (Bridgland and White, 2015).

The gravel lithology and elevation of the deposits make it possible that the site correlates with the Medway Binney Gravels (MIS8-6) or Stoke Gravels (MIS10-8; Bridgland and White, 2015). Bridgland (2003) tentatively advocated for correlation to the latter possibility, based on the findings that Levallois technology was reported in the upper sediments of the initial gravel pits (Tester, 1965, cited by Bridgland and White, 2015). Subsequent work elsewhere at Cuxton in the 1980s also found a typologically similar handaxe assemblage was underlain by a core-and-flake assemblage, including an abundance of flake tools (Cruse, 1987, cited by Wenban-Smith, 2004). This would make a potential Mode 1-2-3 succession at Cuxton, very similar to that seen at Purfleet in MIS10-8, and may support correlation to the earlier Stoke Gravels (Bridgland, 2003; Bridgland and White, 2015).

Nonetheless, typological information is a sub-optimal way of correlating sites, and recent OSL dates of 232.64 ± 13.75 ka and 197.54 ± 17.09 ka at Cuxton has suggested an MIS7 age for the archaeological assemblage (Schwenninger et al., 2007; Wenban-Smith et al., 2007). This reduces age estimates outside those feasible from the Stoke Gravels, and thus may support assignment to the later Binney Gravels. However, the research group that carried out the OSL dating suggest there may have been post-depositional disturbance that reset the bleaching of the quartz grains, resulting in an underestimate of the age (Bates et al., 2014). They therefore suggest that MIS8 is the best correlation for the site, alongside the site of Harnham, Wiltshire (Bates et al., 2014).

The handaxes of Cuxton are known for their overall pointed morphology (Roe's Group I; Roe, 1968), which can be massive in size, and occur amongst a collection of cleavers that is unusual for the British record (Bridgland and White, 2015; Wenban-Smith, 2004). The sample used in the present study was selected from handaxes only amongst the 214 total bifaces documented in the Marshall et al. (2002) database.

**Supplementary References**


Alperson-Afil, N., Goren-Inbar, N., 2010. *The Acheulian Site of Gesher Benot Ya'aqov Volume II: Ancient Flames and Controlled Use of Fire*. Springer Netherlands, Dordrecht.

Alperson-Afil, N., Goren-Inbar, N., 2016. Acheulian hafting: Proximal modification of small flint flakes at Gesher Benot Ya'aqov, Israel. *Quaternary International* 411, 34–43.

Antoine, P., Limondin-Lozouet, N., 2004. Identification of MIS 11 Interglacial tufa deposit in the Somme valley (France): new results from the Saint-Acheul fluvial sequence. *Quaternaire* 15, 41–52.

Antoine, P., Moncel, M.-H., Limondin-Lozouet, N., Locht, J.-L., Bahain, J.-J., Moreno, D., Voinchet, P., Auguste, P., Stoetzel, E., Dabkowski, J., Bello, S.M., Parfitt, S.A., Tombret, O., Hardy, B., 2016. Palaeoenvironment and dating of the Early Acheulean localities from the Somme River basin (Northern France): New discoveries from the High Terrace at Abbeville-Carrière Carpentier. *Quaternary Science Reviews* 149, 338–371.

Antoine, P., Moncel, M.-H., Voinchet, P., Locht, J.-L., Amselem, D., Hérisson, D., Hurel, A., Bahain, J.-J., 2019. The earliest evidence of Acheulian occupation in Northwest Europe and the rediscovery of the Moulin Quignon site, Somme valley, France. *Scientific Reports* 9, 13091.

Ashton, N.M., 1992. The High Lodge flint industries, in: Ashton, N.M., Cook, J., Lewis, S.G., Rose, J. (Eds.), *High Lodge: Excavations by G. de G. Sieveking, 1962-8 and J. Cook, 1988*. British Museum Press, London, pp. 124–163.

Ashton, N.M., Cook, J., Lewis, S.G., Rose, J., 1992. *High Lodge: Excavations by G. de G. Sieveking, 1962-8 and J. Cook, 1988*. British Museum Press, London.

Ashton, N.M., Lewis, S.G., 2005. Maidscross Hill, Lakenheath. *Proceedings of the Suffolk Institute of Archaeology and Natural History* XLI, 122–123.

Ashton, N.M., Lewis, S.G., 2012. The environmental contexts of early human occupation of northwest Europe: The British Lower Palaeolithic record. *Quaternary International* 271, 50–64.

Ashton, N.M., Lewis, S., Parfitt, S., Candy, I., Keen, D., Kemp, R., Penkman, K., Thomas, G., Whittaker, J., White, M., 2005. Excavations at the Lower Palaeolithic site at Elveden, Suffolk, UK. *Proceedings of the Prehistoric Society* 71, 1–61.

Bahain, J.-J., Laurent, M., Falguères, C., Voinchet, P., Farkh, S., Tissoux, H., 2001. Datation par résonnance paramagnétique électronique (RPE) des formations fluviatiles pléistocènes et des gisements archéologiques ou paléontologiques associés. *Quaternaire* 13, 91–103.

Barham, L., Tooth, S., Duller, G.A.T., Plater, A.J., Turner, S., 2015. Excavations at Site C North, Kalambo Falls, Zambia: New Insights into the Mode 2/3 Transition in South-Central Africa. *Journal of African Archaeology* 13, 187–214.

Bates, M.R., Wenban-Smith, F.F., Bello, S.M., Bridgland, D.R., Buck, L.T., Collins, M.J., Keen, D.H., Leary, J., Parfitt, S.A., Penkman, K., Rhodes, E., Ryssaert, C., Whittaker, J.E., 2014. Late persistence of the Acheulian in southern Britain in an MIS 8 interstadial: evidence from Harnham, Wiltshire. *Quaternary Science Reviews* 101, 159–176.

Bridgland, D.R., 2003. The evolution of the River Medway, SE England, in the context of Quaternary palaeoclimate and the Palaeolithic occupation of NW Europe. *Proceedings of the Geologists' Association* 114, 23–48.

Bridgland, D.R., Lewis, S.G., Wymer, J.J., 1995. Middle Pleistocene stratigraphy and archaeology around Mildenhall and Icklingham, Suffolk: report on the Geologists' Association Field Meeting, 27 June, 1992. *Proceedings of the Geologists' Association* 106, 57–69.

Bridgland, D.R., White, M.J., 2015. Chronological variations in handaxes: patterns detected from fluvial archives in north-west Europe. *Journal of Quaternary Science* 30, 623–638.

Brumm, A., McLaren, A., 2011. Scraper reduction and "imposed form" at the Lower Palaeolithic site of High Lodge, England. *Journal of Human Evolution* 60, 185–204.

Callow, P., 1976. The Lower and Middle Palaeolithic of Britain and Adjacent Areas of Europe (Unpublished PhD Dissertation). University of Cambridge, Cambridge.

Caton-Thompson, G., 1952. *Kharga Oasis in Prehistory*. The Athlone Press, London.

Chandler, R.H., Leach, A.L., 1912. On the Dartford Heath gravel and on a Palaeolithic implement factory. *Proceedings of the Geologists' Association* 23, 102–111.

Chedeville, P., 1896. Recherches préhistoriques and géologiques sur la station paléolithique et dépôt quaternaire ou pléistocène de Saint-Pierre-lès Elbeuf. *Bulletin de la Société d'étude des sciences naturelles d'Elbeuf* 14.

Churcher, C.S., Kleindienst, M.R., Schwarcz, H.P., 1999. Faunal remains from a Middle Pleistocene lacustrine marl in Dakhleh Oasis, Egypt: palaeoenvironmental reconstructions. *Palaeogeography, Palaeoclimatology, Palaeoecology* 154, 301–312.

Clark, J.D., 2001. *Kalambo Falls Prehistoric Site: Volume III. The Earlier Cultures: Middle and Earlier Stone Age*. Cambridge University Press, Cambridge.

Cliquet, D., Lautridou, J.-P., Antoine, P., Lamothe, M., Leroyer, M., Limondin-Lozouet, N., Mercier, N., 2009. La séquence loessique de Saint-Pierre-lès-Elbeuf (Normandie, France): nouvelles données archéologiques, géochronologiques et paléontologiques. *Quaternaire* 321–343.

Coe, R.S., Singer, B.S., Pringle, M.S., Zhao, X., 2004. Matuyama–Brunhes reversal and Kamikatsura event on Maui: paleomagnetic directions, 40Ar/39Ar ages and implications. *Earth and Planetary Science Letters* 222, 667–684.

Commont, V., 1909. Saint-Acheul et Montieres Notes de Géologie, de Paléontologie et de Préhistoire. *Mémoires de la Société Geologique du Nord* III, 68.

Commont, V., 1911. Les gisements préhistoriques de Saint-Acheul et de Montures. *Notes de Préhistoire publiées dans le Bulletin de la Société Lineenne du Nord de la France de 1905 à 1910*. Amiens.

Conway, B., 1996. The geology outside the National Nature Reserve, 1968-72, in: Conway, B., McNabb, J., Ashton, N. (Eds.), *Excavations at Barnfield Pit, Swanscombe, 1968–72, British Museum Occasional Paper*. British Museum Press, London, pp. 67–88.

Conway, B., McNabb, J., Ashton, N., 1996. *Excavations at Barnpit Field, Swanscombe, 1968–72, British Museum Occasional Paper*. British Museum Press, London.

Coope, G.R., 1992. The High Lodge insect fauna, in: Ashton, N.M., Cook, J., Lewis, S.G., Rose, J. (Eds.), *High Lodge: Excavations by G. de G. Sieveking, 1962-8 and J. Cook, 1988*. British Museum Press, London, pp. 117–119.

Costa, A.G., 2010. A Geometric Morphometric Assessment of Plan Shape in Bone and Stone Acheulean Bifaces from the Middle Pleistocene Site of Castel di Guido, Latium, Italy, in: Lycett, S., Chauhan, P. (Eds.), *New Perspectives on Old Stones: Analytical Approaches to Paleolithic Technologies*. Springer US, New York, NY, pp. 23–41.

Cruse, R.J., 1987. Further investigation of the Acheulian site at Cuxton. *Archaeologia Cantiana* 104, 39–81.

Davis, R., Ashton, N.M., 2019. Landscapes, environments and societies: The development of culture in Lower Palaeolithic Europe. *Journal of Anthropological Archaeology* 56, 101107.

Davis, R.J., Lewis, S.G., Ashton, N.M., Parfitt, S.A., Hatch, M.T., Hoare, P.G., 2017. The early Palaeolithic archaeology of the Breckland: current understanding and directions for future research. *The Journal of Breckland Studies* 1, 28–44.

de Mortillet, G., 1872. Classification des diverses périodes de l'âge de la pierre. Presented at the Congrès international d'Anthropologie et d'Archéologie préhistoriques, 6ème session, Bruxelles, 1872, C. Muquardt, Brussels, pp. 432–459.

Deino, A.L., 2012. 40Ar/39Ar dating of Bed I, Olduvai Gorge, Tanzania, and the chronology of early Pleistocene climate change. *Journal of Human Evolution* 63, 251–273.

Deino, A.L., Heil, C., King, J., McHenry, L.J., Stanistreet, I.G., Stollhofen, H., Njau, J.K., Mwankunda, J., Schick, K.D., Toth, N., 2021. Chronostratigraphy and age modeling of

Pleistocene drill cores from the Olduvai Basin, Tanzania (Olduvai Gorge Coring Project). *Palaeogeography, Palaeoclimatology, Palaeoecology* 571, 109990.

Deino, A.L., McBrearty, S., 2002. 40Ar/39Ar dating of the Kapthurin Formation, Baringo, Kenya. *Journal of Human Evolution* 42, 185–210.

Deino, A.L., Potts, R., 1990. Single-crystal 40Ar/39Ar dating of the Olorgesailie Formation, Southern Kenya Rift. *Journal of Geophysical Research: Solid Earth* 95, 8453–8470.

Despriée, J., Courcimault, G., Moncel, M.-H., Voinchet, P., Tissoux, H., Puaud, S., Gallet, X., Bahain, J.-J., Moreno, D., Falguères, C., 2016. The Acheulean site of la Noira (Centre region, France): Characterization of materials and alterations, choice of lacustrine millstone and evidence of anthropogenic behaviour. *Quaternary International* 411, 144–159.

Despriée, J., Voinchet, P., Tissoux, H., Bahain, J.-J., Falguères, C., Courcimault, G., Dépont, J., Moncel, M.-H., Robin, S., Arzarello, M., Sala, R., Marquer, L., Messager, E., Puaud, S., Abdessadok, S., 2011. Lower and Middle Pleistocene human settlements recorded in fluvial deposits of the middle Loire River Basin, Centre Region, France. *Quaternary Science Reviews* 30, 1474–1485.

di Cesnola, A.P., Mallegni, F., 1996. *Le Paléolithique inférieur et moyen en Italie*. Editions Jérôme Millon, Grenoble.

Diez-Martín, F., Sánchez Yustos, P., Uribelarrea, D., Baquedano, E., Mark, D.F., Mabulla, A., Fraile, C., Duque, J., Díaz, I., Pérez-González, A., Yravedra, J., Egeland, C.P., Organista, E., Domínguez-Rodrigo, M., 2015. The Origin of The Acheulean: The 1.7 Million-Year-Old Site of FLK West, Olduvai Gorge (Tanzania). *Scientific Reports* 5, 17839.

Domínguez-Rodrigo, M., 2009. Are all Oldowan Sites Palimpsests? If so, what can they tell us about Hominid Carnivory?, in: Hovers, E., Braun, D.R. (Eds.), *Interdisciplinary Approaches to the Oldowan*. Springer Netherlands, Dordrecht, pp. 129–147.

Domínguez-Rodrigo, M., Pickering, T.R., Baquedano, E., Mabulla, A., Mark, D.F., Musiba, C., Bunn, H.T., Uribelarrea, D., Smith, V., Diez-Martin, F., Pérez-González, A., Sánchez, P., Santonja, M., Barboni, D., Gidna, A., Ashley, G., Yravedra, J., Heaton, J.L., Arriaza, M.C., 2013. First Partial Skeleton of a 1.34-Million-Year-Old Paranthropus boisei from Bed II, Olduvai Gorge, Tanzania. *PLoS ONE* 8, e80347.

Duller, G.A.T., Tooth, S., Barham, L., Tsukamoto, S., 2015. New investigations at Kalambo Falls, Zambia: Luminescence chronology, site formation, and archaeological significance. *Journal of Human Evolution* 85, 111–125.

Durkee, H., Brown, F.H., 2014. Correlation of volcanic ash layers between the Early Pleistocene Acheulean sites of Isinya, Kariandusi, and Olorgesailie, Kenya. *Journal of Archaeological Science* 49, 510–517.

Duval, M., Voinchet, P., Arnold, L.J., Parés, J.M., Minnella, W., Guilarte, V., Demuro, M., Falguères, C., Bahain, J.-J., Despriée, J., 2020. A multi-technique dating study of two Lower Palaeolithic sites from the Cher Valley (Middle Loire Catchment, France): Lunery-la Terre-des-Sablons and Brinay-la Noira. *Quaternary International* 556, 79–95.

Feibel, C.S., 2004. Quaternary lake margins of the Levant Rift Valley, in: Goren-Inbar, N., Speth, J.D. (Eds.), *Human Paleocology in the Levantine Corridor*. Oxbow Books, Oxford, pp. 21–36.

Flower, J.W., 1869. On some recent discoveries of flint implements of the Drift in Norfolk and Suffolk., with observations of theories accounting for their distribution. *Quarterly Journal of the Geological Society of London* 25, 449–460.

García-Medrano, P., Ollé, A., Ashton, N., Roberts, M.B., 2019. The Mental Template in Handaxe Manufacture: New Insights into Acheulean Lithic Technological Behavior at Boxgrove, Sussex, UK. *Journal of Archaeological Method and Theory* 26, 396–422.

García-Medrano, P., Despriée, J., Moncel, M.-H., 2022. Innovations in Acheulean biface production at la Noira (France) during Middle Pleistocene in Western Europe. *Archaeological and Anthropological Sciences* 14, 69.

Garrod, D.A.E., Bate, D.M.A., 1937. *The Stone Age of Mount Carmel Vol. I*. Clarendon Press, Oxford.

Gibbard, P.L., Pasanen, A.H., West, R.G., Lunkka, J.P., Boreham, S., Cohen, K.M., Rolfe, C., 2009. Late Middle Pleistocene glaciation in East Anglia, England. *Boreas* 38, 504–528.

Goren-Inbar, N., 2017. Gesher Benot Ya'aqov, in: Enzel, Y., Bar-Yosef, O. (Eds.), *Quaternary of the Levant: Environments, Climate Change, and Humans*. Cambridge University Press, Cambridge, pp. 187–194.

Goren-Inbar, N., Alperson-Afil, N., Sharon, G., Herzlinger, G., 2018. *The Acheulian Site of Gesher Benot Ya'aqov Volume IV: The Lithic Assemblages*. Springer International Publishing, Cham.

Goren-Inbar, N., Feibel, C.S., Verosub, K.L., Melamed, Y., Kislev, M.E., Tchernov, E., Saragusti, I., 2000. Pleistocene Milestones on the Out-of-Africa Corridor at Gesher Benot Ya'aqov, Israel. *Science* 289, 944–947.

Goren-Inbar, N., Kislev, M.E., Melamed, Y., Rabinovich, R., Zohar, I., Biton, R., et al., 2012. The effect of climate change on the environment and hominins of the Upper Jordan Valley between ca. 800Ka and 700Ka ago as a basis for prediction of future scenarios. Hebrew University (Israel Science Foundation Grant), Jerusalem.

Gowlett, J.A.J., 1988. A case of Developed Oldowan in the Acheulean? *World Archaeology* 20, 13–26.

Gowlett, J.A.J., Crompton, R.H., 1994. Kariandusi: Acheulean morphology and the question of allometry. *African Archaeological Review* 12, 3–42.

Green, C.P., 1988. The Palaeolithic site at Broom, Dorset, 1932–41: from the record of C.E. Bean, Esq., F.S.A. *Proceedings of the Geologists' Association* 99, 173–180.

Green, C.P., Hosfield, R.T., 2013. *Quaternary History and Palaeolithic Archaeology in the Axe Valley at Broom, South West England*. Oxbow Books, Oxford.

Grün, R., Stringer, C., 2000. Tabun revisited: revised ESR chronology and new ESR and U-series analyses of dental material from Tabun C1. *Journal of Human Evolution* 39, 601–612.

Haahr, M., 2020. RANDOM.ORG: True Random Number Service. URL: https://www.random.org

Hardaker, T., Dunn, S., 2005. The flip test–a new statistical measure for quantifying symmetry in stone tools. *Antiquity* 79(306).

Hardy, B.L., Moncel, M.-H., Despriée, J., Courcimault, G., Voinchet, P., 2018. Middle Pleistocene hominin behavior at the 700ka Acheulean site of la Noira (France). *Quaternary Science Reviews* 199, 60–82.

Hay, R.L., 1976. *Geology of the Olduvai Gorge: A Study of Sedimentation in a Semiarid Basin*. University of California Press, Berkeley.

Hay, R.L., 1990. Olduvai Gorge: a case history in the interpretation of hominid paleoenvironments in East Africa, in: Laporte, L.F. (Ed.), *Establishment of a Geological Framework for Paleoanthropology*. Geological Society of America, Boulder, CO, pp. 23–37.

Herzlinger, G., Goren-Inbar, N., 2019. Do a few tools necessarily mean a few people? A techno-morphological approach to the question of group size at Gesher Benot Ya'aqov, Israel. *Journal of Human Evolution* 128, 45–58.

Herzlinger, G., Goren-Inbar, N., 2020. Beyond a Cutting Edge: a Morpho-technological Analysis of Acheulian Handaxes and Cleavers from Gesher Benot Yaʻaqov, Israel. *Journal of Paleolithic Archeology* 3, 33–58.

Herzlinger, G., Grosman, L., 2018. AGMT3-D: A software for 3-D landmarks-based geometric morphometric shape analysis of archaeological artifacts. *PLoS ONE* 13, e0207890.

Herzlinger, G., Wynn, T., Goren-Inbar, N., 2017. Expert cognition in the production sequence of Acheulian cleavers at Gesher Benot Ya'aqov, Israel: A lithic and cognitive analysis. *PLoS ONE* 12, e0188337.

Hosfield, R.T., Chambers, J.C., 2004. *The Archaeological Potential of Secondary Contexts. (English Heritage Archive Report No. 3361)*. English Heritage, London.

Hosfield, R.T., Chambers, J.C., 2009. Genuine Diversity? The Broom Biface Assemblage. *Proceedings of the Prehistoric Society* 75, 65–100.

Hunt, C.O., 1992. Pollen and algal microfossils from the High Lodge clayey-silts, in: Ashton, N.M., Cook, J., Lewis, S.G., Rose, J. (Eds.), *High Lodge: Excavations by G. de G. Sieveking, 1962-8 and J. Cook, 1988*. British Museum Press, London, pp. 109–115.

IBM Corp, 2019. IBM SPSS Statistics for Windows, Version 26.0. Armonk, NY: IBM Corp.

Iovita, R., 2009. Ontogenetic scaling and lithic systematics: method and application. *Journal of Archaeological Science* 36, 1447–1457.

Iovita, R., McPherron, S.P., 2011. The handaxe reloaded: A morphometric reassessment of Acheulian and Middle Paleolithic handaxes. *Journal of Human Evolution* 61, 61–74.

Iovita, R., Tuvi-Arad, I., Moncel, M.-H., Despriée, J., Voinchet, P., Bahain, J.-J., 2017. High handaxe symmetry at the beginning of the European Acheulian: The data from la Noira (France) in context. *PLoS ONE* 12, e0177063.

Isaac, G.L., 1969. Studies of early culture in East Africa. *World Archaeology* 1, 1–28.

Isaac, G.L., 1977. *Olorgesailie: Archaeological Studies of a Middle Pleistocene Lake Basin in Kenya*. University of Chicago Press, Chicago, IL.

Jagher, R., 2011. Nadaouiyeh Aïn Askar - Acheulean Variability in the Central Syrian Desert, in: The Lower and Middle Palaeolthic in the Middle East and Neighbouring Regions. Presented at the Basel Symposium (May 8-10 2008), ERAUL, Liège, pp. 209–224.

Jagher, R., 2016. Nadaouiyeh Aïn Askar, an example of Upper Acheulean variability in the Levant. *Quaternary International* 411, 44–58.

Jelinek, A.J., 1982a. The Tabun Cave and Paleolithic Man in the Levant. *Science* 216, 1369–1375.

Jelinek, A.J., 1982b. The Middle Paleolithic in the southern Levant with comments on the appearance of modern Homo Sapiens, in: Ronen, A. (Ed.), *The Transition from Lower to Middle Paleolithic and the Origin of Modern Man, BAR International Series*. British Archaeological Reports, Oxford, pp. 57–101.

Johnson, C.C., Njau, J.K., Damme, D.V., Schick, K., Toth, N., 2016. Palaeoecologic Significance of Malacofauna, Olduvai Gorge, Tanzania. *PALAIOS* 31, 319–326.

Kalbe, J., Jagher, R., Pümpin, C., 2016. The spring of Nadaouiyeh Aïn Askar — Paleoecology of a Paleolithic oasis in arid central Syria. *Palaeogeography, Palaeoclimatology, Palaeoecology* 446, 252–262.

Kleindienst, M.R., Schwarcz, H.P., Nicoll, K.A., Churcher, C.S., Frizano, J., Giegengack, R., Wiseman, M.F., 2008. Water in the Desert: First Report on Uranium-series Dating of Caton-Thompson's and Gardner's 'Classic' Pleistocene Sequence at Refuf Pass, Kharga Oasis, in: Wiseman, M.F. (Ed.), *The Oasis Papers 2. Proceedings of the Second International Conference of the Dakhleh Oasis Project, Dakhleh Oasis Project*. Oxbow Books, Oxford.

Laukhin, S.A., Ronen, A., Ranov, V.A., et al., 2000. New data on the Paleolithic geochronology in southern Levant. *Stratigraphy and Geological Correlation* 8, 498–510.

Le Tensorer, J.-M., 2006. Les cultures acheuléennes et la question de l'émergence de la pensée symbolique chez Homo erectus à partir des données relatives à la forme symétrique et harmonique des bifaces. *Comptes Rendus Palevol* 5, 127–135.

Le Tensorer, J.-M., Jagher, R., Rentzel, P., Hauck, T., Ismail-Meyer, K., Pümpin, C., Wojtczak, D., 2007. Long-term site formation processes at the natural springs Nadaouiyeh and Hummal in the El Kowm Oasis, Central Syria. *Geoarchaeology* 22, 621–640.

Leach, A.L., 1913. On buried channels in the Dartford Heath Gravel: Together with the report of an excursion to Dartford Heath, May 17th, 1913. *Proceedings of the Geologists' Association* 24, 337–344.

Leakey, L.S.B., 1951. *Olduvai Gorge: A report on the evolution of the hand-axe culture in Beds I-IV. With chapters on the geology and fauna*. Cambridge University Press, Cambridge.

Leakey, M.D., 1971. *Olduvai Gorge: Volume III, Excavations in Beds I and II, 1960-1963*. Cambridge University Press, Cambridge.

Leakey, M.D., Roe, D.A., 1994. *Olduvai Gorge Volume V: Excavations in Beds III, IV and the Masek Beds, 1968–1971*. Cambridge University Press, Cambridge.

Leakey, M.D., Tobias, P.V., Martyn, J.E., Leakey, R.E.F., 1969. An Acheulean Industry with Prepared Core Technique and the Discovery of a Contemporary Hominid Mandible at Lake Baringo, Kenya. *Proceedings of the Prehistoric Society* 35, 48–76.

Lee, C., Bada, J.L., Peterson, E., 1976. Amino acids in modern and fossil woods. *Nature* 259, 183–186.

Lee, J.R., Rose, J., Hamblin, R.J.O., Moorlock, B.S.P., 2004. Dating the earliest lowland glaciation of eastern England: a pre-MIS 12 early Middle Pleistocene Happisburgh glaciation. *Quaternary Science Reviews* 23, 1551–1566.

Lefèvre, D., Raynal, J.-P., Vernet, G., Kieffer, G., Piperno, M., 2010. Tephro-stratigraphy and the age of ancient Southern Italian Acheulean settlements: The sites of Loreto and Notarchirico (Venosa, Basilicata, Italy). *Quaternary International* 223–224, 360–368.

Leroyer, M., Cliquet, D., 2010. Continuity or discontinuity of the ‘Acheulean tradition’ through the middle Pleistocene? The example of the Mont-Enot site at Saint-Pierre-lès-Elbeuf, Seine-Maritime, France. *Quaternary International* 223–224, 462–464.

Lewis, S.G., 1992. High Lodge – stratigraphy and depositional environments, in: Ashton, N.M., Cook, J., Lewis, S.G., Rose, J. (Eds.), *High Lodge: Excavations by G. de G. Sieveking, 1962-8 and J. Cook, 1988*. British Museum Press, London, pp. 51–93.

Limondin-Lozouet, N., Antoine, P., 2006. A new Lyrodiscus (Mollusca, Gastropoda) assemblage from Saint-Acheul (Somme Valley): a reappraisal of MIS 11 malacofaunas from northern France. *Boreas* 35, 622–633.

Limondin-Lozouet, N., Nicoud, E., Antoine, P., Auguste, P., Bahain, J.-J., Dabkowski, J., Dupéron, J., Dupéron, M., Falguères, C., Ghaleb, B., Jolly-Saad, M.-C., Mercier, N., 2010. Oldest evidence of Acheulean occupation in the Upper Seine valley (France) from an MIS 11 tufa at La Celle. *Quaternary International* 223–224, 299–311.

Macphail, R.I., 1999. Sediment micromorphology, in: *Boxgrove: A Middle Pleistocene Hominid Site at Eartham Quarry, Boxgrove, West Sussex*. English Heritage, London, pp. 118–148.

Marshall, G.D., Gamble, C.G., Roe, D.A., and Dupplaw, D. 2002. Acheulian biface database. Archaeology Data Service, York. URL: http://archaeologydataservice.ac.uk/archives/view/bifaces/bf_query.cfm.

McBrearty, S., Tryon, C., 2005. From Acheulean to Middle Stone Age in the Kapthurin Formation, Kenya, in: Hovers, E., Kuhn, S.L. (Eds.), *Transitions Before the Transition: Evolution and Stability in the Middle Paleolithic and Middle Stone Age*. Springer US, Boston, MA, pp. 257–277.

McHenry, L.J., Stanistreet, I.G., 2018. Tephrochronology of Bed II, Olduvai Gorge, Tanzania, and placement of the Oldowan–Acheulean transition. *Journal of Human Evolution* 120, 7–18.

McKinney, C., 2001. The uranium-series age of wood from Kalambo Falls, in: *Kalambo Falls Prehistoric Site: Volume III. The Earlier Cultures: Middle and Earlier Stone Age*. Cambridge University Press, London, pp. 665–674.

McPherron, S.P., 2003. Technological and Typological Variability in the Bifaces from Tabun Cave, Israel, in: Soressi, M., Dibble, H.L. (Eds.), *Multiple Approaches to the Study of Bifacial Technologies*. University of Pennsylvania, Museum of Archaeology and Anthropology, Philadelphia, PA, pp. 55–75.

McPherron, S.P., Dibble, H.L., 1999. Stone Tool Analysis Using Digitized Images: Examples from the Lower and Middle Paleolithic. *Lithic Technology* 24, 38–52.

Mercier, N., Valladas, H., 2003. Reassessment of TL age estimates of burnt flints from the Paleolithic site of Tabun Cave, Israel. *Journal of Human Evolution* 45, 401–409.

Mercier, N., Valladas, H., Froget, L., Joron, J.-L., Ronen, A., 2000. Datation par thermoluminescence de la base du gisement paléolithique de Tabun (mont Carmel, Israël). *Comptes Rendus de l'Académie des Sciences - Series IIA - Earth and Planetary Science* 330, 731–738.

Merrick, H.V., Brown, F.H., Nash, W.P., 1994. Use and movement of obsidian in the Early and Middle Stone Ages of Kenya and northern Tanzania. *Society, Culture, and Technology in Africa*, 29–44.

Moncel, M.-H., Antoine, P., Herisson, D., Locht, J.-L., Hurel, A., Bahain, J.-J., 2022. Were Hominins Specifically Adapted to North-Western European Territories Between 700 and 600 ka? New Insight Into the Acheulean Site of Moulin Quignon (France, Somme Valley). *Frontiers in Earth Science* 10, 882110.

Moncel, M.-H., Ashton, N., 2018. From 800 to 500 ka in Western Europe. The Oldest Evidence of Acheuleans in Their Technological, Chronological, and Geographical Framework, in: Gallotti, R., Mussi, M. (Eds.), *The Emergence of the Acheulean in East Africa and Beyond*. Springer International Publishing, Cham, pp. 215–235.

Moncel, M.-H., Ashton, N., Lamotte, A., Tuffreau, A., Cliquet, D., Despriée, J., 2015. The Early Acheulian of north-western Europe. *Journal of Anthropological Archaeology* 40, 302–331.

Moncel, M.-H., Despriée, J., Courcimaut, G., Voinchet, P., Bahain, J.-J., 2020a. La Noira Site (Centre, France) and the Technological Behaviours and Skills of the Earliest Acheulean in Western Europe Between 700 and 600 ka. *Journal of Paleolithic Archeology* 3, 255–301.

Moncel, M.-H., Despriée, J., Voinchet, P., Courcimault, G., Hardy, B., Bahain, J.-J., Puaud, S., Gallet, X., Falguères, C., 2016. The Acheulean workshop of la Noira (France, 700 ka) in the European technological context. *Quaternary International* 393, 112–136.

Moncel, M.-H., Despriée, J., Voinchet, P., Tissoux, H., Moreno, D., Bahain, J.-J., Courcimault, G., Falguères, C., 2013. Early Evidence of Acheulean Settlement in Northwestern Europe - La Noira Site, a 700,000-Year-Old Occupation in the Center of France. *PLoS ONE* 8, e75529.

Moncel, M.-H., Lemorini, C., Eramo, G., Fioretti, G., Daujeard, C., Curci, A., Berto, C., Hardy, B., Pineda, A., Rineau, V., Carpentieri, M., Sala, B., Arzarello, M., Mecozzi, B., Iannucci, A., Sardella, R., Piperno, M., 2023. A taphonomic and spatial distribution study of the new levels of the middle Pleistocene site of Notarchirico (670–695 ka, Venosa, Basilicata, Italy). *Archaeological and Anthropological Sciences* 15, 106.

Moncel, M.-H., García-Medrano, P., Despriée, J., Arnaud, J., Voinchet, P., Bahain, J.-J., 2021. Tracking behavioral persistence and innovations during the Middle Pleistocene in Western Europe. Shift in occupations between 700 and 450 ka at la Noira site (Centre, France). *Journal of Human Evolution* 156, 103009.

Moncel, M.-H., Santagata, C., Pereira, A., Nomade, S., Bahain, J.-J., Voinchet, P., Piperno, M., 2019. A biface production older than 600 ka ago at Notarchirico (Southern Italy) contribution to understanding early Acheulean cognition and skills in Europe. *PLoS ONE* 14, e0218591.

Moncel, M.-H., Santagata, C., Pereira, A., Nomade, S., Voinchet, P., Bahain, J.-J., Daujeard, C., Curci, A., Lemorini, C., Hardy, B., Eramo, G., Berto, C., Raynal, J.-P., Arzarello, M., Mecozzi, B., Iannucci, A., Sardella, R., Allegretta, I., Delluniversità, E., Terzano, R., Dugas, P., Jouanic, G., Queffelec, A., d'Andrea, A., Valentini, R., Minucci, E., Carpentiero, L., Piperno, M., 2020b. The origin of early Acheulean expansion in Europe 700 ka ago: new findings at Notarchirico (Italy). *Scientific Reports* 10, 13802.

Newton, E.T., 1895. On the human skull and limb-bones found in the Palaeolithic Terrace Gravel at Galley Hill, Kent. *Quarterly Journal of the Geological Society of London* 51, 505–527.

Njau, J.K., Herrmann, E.W., Ruck, L., Pante, M., Farrugia, P., Toth, N., Schick, K., Stollhofen, H., Stanistreet, I.G., 2020. Core stratigraphy constrains Bed IV archaeological record at HEB site, Olduvai Gorge, Tanzania. *Palaeogeography, Palaeoclimatology, Palaeoecology* 552, 109773.

Nyamweru, C., 1980. *Rifts and Volcanoes: a study of the East African rift system*. Nelson, Nairobi.

Ovey, C.D., 1964. *The Swanscombe Skull. A Survey of Research on a Pleistocene Site, Royal Anthropological Institute Occasional Paper*. Royal Anthropological Institue of Great Britain and Ireland, London.

Owen, R.B., Potts, R., Behrensmeyer, A.K., Ditchfield, P., 2008. Diatomaceous sediments and environmental change in the Pleistocene Olorgesailie Formation, southern Kenya Rift Valley. *Palaeogeography, Palaeoclimatology, Palaeoecology* 269, 17–37.

Pereira, A., Nomade, S., Voinchet, P., Bahain, J.J., Falguères, C., Garon, H., Lefèvre, D., Raynal, J.P., Scao, V., Piperno, M., 2015. The earliest securely dated hominin fossil in Italy and evidence of Acheulian occupation during glacial MIS 16 at Notarchirico (Venosa, Basilicata, Italy). *Journal of Quaternary Science* 30, 639–650.

Pillyere, T., Sanzelle, S., Fain, S., et al., 1999. Essai de datation par thermoluminescence des dépôts du site acheuléen de Notarchirico, in: Piperno, M. (Ed.), *Notarchirico: Un Sito Del Pleistocene Medio-antico Nel Bacino Di Venosa (Basilicata)*. Osanna edition, Venosa, pp. 235–243.

Piperno, M., 1999. *Notarchirico: Un sito del Pleistocene medio-antico nel bacino di Venosa (Basilicata)*. Osanna Edition, Venosa.

Piperno, M., Mallegni, F., Yokoyama, Y., 1990. Découverte d'un fémur humain dans les niveaux acheuléens de Notarchirico (Venosa, Basilicata, Italie). *Comptes rendus de l'Académie des sciences*. 311, 1097–1102.

Piperno, M., Tagliacozzo, A., 2001. The Elephant Butchery Area at the Middle Pleistocene site of Notarchirico (Venosa, Basilicata, Italy), in: Cavarretta, G., Gioia, P., Mussi, M., Palombo, M.R. (Eds.), *The World of Elephants: Proceedings of the 1st International Congress*. Consiglio Nazionale delle Ricerche, Roma, pp. 230–236.

Pope, M., Roberts, M.B., 2005. Individuals and artefact scatters at Boxgrove, in: Gamble, C.S., Porr, M. (Eds.), *The Hominid Individual in Context*. Routledge, Abingdon, pp. 81–97.

Potts, R., 1989. Olorgesailie: new excavations and findings in Early and Middle Pleistocene contexts, southern Kenya rift valley. *Journal of Human Evolution* 18, 477–484.

Potts, R., Behrensmeyer, A.K., Deino, A., Ditchfield, P., Clark, J., 2004. Small Mid-Pleistocene Hominin Associated with East African Acheulean Technology. *Science* 305, 75–78.

Potts, R., Behrensmeyer, A.K., Ditchfield, P., 1999. Paleolandscape variation and Early Pleistocene hominid activities: Members 1 and 7, Olorgesailie Formation, Kenya. *Journal of Human Evolution* 37, 747–788.

Prestwich, J., 1860. On the occurrence of flint implements associated with remains of animals of extinct species in beds of a late geological period in France at Amiens and Abbeville and in England at Hoxne. *Philosophical Transactions of the Royal Society of London* 150, 277–327.

Proffitt, T., 2018. Is there a Developed Oldowan A at Olduvai Gorge? A diachronic analysis of the Oldowan in Bed I and Lower-Middle Bed II at Olduvai Gorge, Tanzania. *Journal of Human Evolution* 120, 92–113.

Pümpin, C., 2003. Geoarchäologische Untersuchungen an der pleistozänen Fundstelle von Nadaouiyeh Aïn Askar (Syrien). Unpublished M.A. Thesis. Universität Basel, Basel.

Raynal, J.P., Lefèvre, D., Vernet, G., Pilleyre, T., Sanzelle, S., Fain, J., Miallier, D., Montret, M., 1998. Sedimentary Dynamics and Tecto-Volcanism in the Venosa Basin (Basilicata, Italia). *Quaternary International* 47–48, 97–105.

Renault-Miskovsky, J., 1998. Etude pollinique du site de Nadaouiyeh Ain Askar (Nad-1, El Kowm, Syrie). Premiers Resultats. (No. 3), Travaux de la Mission Syro-Suisse d'El Kowm.

Reynaud Savioz, N., 2011. The Faunal Remains from Nadaouiyeh Aïn Askar (Syria). Preliminary Indications of Animal Acquisition in an Acheulean Site, in: The Lower and Middle Palaeolthic in the Middle East and Neighbouring Regions. Presented at the Basel Symposium (May 8-10 2008), ERAUL, Liège, pp. 225–233.

Rigollot, M.-J., 1854. Mémoire sur des instruments en silex trouvés à Saint-Acheul, d'Amiens, et considérés sous les rapports géologiques et archéologiques. *Mémoire de la Société des Antiquaires de Picardie* XIV, 22–60.

Rineau, V., Moncel, M.-H., Zeitoun, V., 2023. Revealing Evolutionary Patterns Behind Homogeneity: the Case of the Palaeolithic Assemblages from Notarchirico (Southern Italy). *Journal of Archaeological Method and Theory* 30, 203–238.

Rink, W.J., Schwarcz, H.P., Ronen, A., Tsatskin, A., 2004. Confirmation of a near 400 ka age for the Yabrudian industry at Tabun Cave, Israel. *Journal of Archaeological Science* 31, 15–20.

Roberts, M., Parfitt, S., 1999. *Boxgrove: A Middle Pleistocene Hominid Site at Eartham Quarry, Boxgrove, West Sussex*. English Heritage, London.

Roche, H., Brugal, J.-P., Lefevre, D., Ploux, S., Texier, P.-J., 1988. Isenya: état des recherches sur un nouveau site acheuléen d'Afrique orientale. *African Archaeological Review* 6, 27–55.

Roe, D.A., 1968. British Lower and Middle Palaeolithic Handaxe Groups. *Proceedings of the Prehistoric Society* 34, 1–82.

Roksandic, M., Radović, P., Lindal, J., 2018. Revising the hypodigm of Homo heidelbergensis: A view from the Eastern Mediterranean. *Quaternary International* 466, 66–81.

Ronen, A., 2017. Tabun Cave in the Carmel Culture Sphere, in: Enzel, Y., Bar-Yosef, O. (Eds.), *Quaternary of the Levant: Environments, Climate Change, and Humans*. Cambridge University Press, Cambridge, pp. 215–224.

Ronen, A., Shifroni, A., Laukhin, S.A., Tsatskin, A., 2000. Observations on the Acheulean of Tabun Cave, Israel, in: Mester, Z., Ringer, A. (Eds.), *A La Recherche de L'Homme Préhistorique*. ERAUL, University of Liége Press, Liége, pp. 202–224.

Rose, J., 1992. High Lodge – regional context and geological background, in: Ashton, N.M., Cook, J., Lewis, S.G. (Eds.), *High Lodge: Excavations by G. de G. Sieveking, 1962-8 and J. Cook, 1988*. British Museum Press, London, pp. 13–24.

Sahnouni, M., Semaw, S., Rogers, M., 2013. The African Acheulean, in: Mitchell, P., Lane, P.J. (Eds.), *The Oxford Handbook of African Archaeology*. Oxford University Press.

Sala, B., 1990. A preliminary report on the microvertebrates of Notarchirico, Venosa. (Rapporto preliminare sui microvertebrati di Notarchirico, Venosa). *Preistoria Alpina* 25, 7–14.

Sala, B., 1999. Nuovi dati Sulla microteriofauna di Notarchirico, in: Piperno, M. (Ed.), *Notarchirico: Un Sito Del Pleistocene Medio-antico Nel Bacino Di Venosa (Basilicata)*. Osanna edition, Venosa, pp. 439–441.

Sánchez-Yustos, P., Diez-Martín, F., Díaz, I., Fraile, C., Uribelarrea, D., Mabulla, A., Baquedano, E., Domínguez-Rodrigo, M., 2019. What comes after the Developed Oldowan B debate? Techno-economic data from SHK main site (Middle Bed II, Olduvai Gorge, Tanzania). *Quaternary International* 526, 67–76.

Sánchez-Yustos, P., Diez-Martín, F., Domínguez-Rodrigo, M., Fraile, C., Duque, J., Díaz, I., de Francisco, S., Baquedano, E., Mabulla, A., 2018. Acheulean without handaxes? Assemblage variability at FLK West (Lowermost Bed II, Olduvai, Tanzania). *Journal of Anthropological Sciences* 53–73.

Santagata, C., 2016. Operating systems in units B and E of the Notarchirico (Basilicata, Italy) ancient Acheulean open-air site and the role of raw materials. *Quaternary International* 411, 284–300.

Schick, K.D., 2001. An examination of Kalambo Falls Acheulean Site B5 from a geoarchaeological perspective, in: *Kalambo Falls Prehistoric Site: Volume III. The Earlier Cultures: Middle and Earlier Stone Age*. Cambridge University Press, Cambridge, pp. 463–480.

Schmid, P., 2015. Nadaouiyeh – A Homo erectus in Acheulean context. *L'Anthropologie* 119, 694–705.

Schreve, D.C., 2001. Mammalian evidence from Middle Pleistocene fluvial sequences for complex environmental change at the oxygen isotope substage level. *Quaternary International* 79, 65–74.

Schwenninger, J.-L., Wenban-Smith, F., Bates, M.R., Briant, R.M., 2007. Medway Valley Palaeolithic Project Final Report: The Palaeolithic Resource in the Medway Gravels (Kent). Appendix 6. Optically stimulated luminescence (OSL) dating of fluvial sediments from the Medway Valley. English Heritage, London.

Semaw, S., Rogers, M., Stout, D., 2009. The Oldowan-Acheulian Transition: Is there a "Developed Oldowan" Artifact Tradition?, in: Camps, M., Chauhan, P. (Eds.), *Sourcebook of Paleolithic Transitions: Methods, Theories, and Interpretations*. Springer New York, New York, NY, pp. 173–193.

Shakesby, R.A., Stephens, N., 1984. The Pleistocene gravels of Axe Valley, Devon. *Report of the Transactions of the Devon Association for the Advancement of Science* 116, 77–88.

Sharon, G., 2007. *Acheulian Large Flake Industries: Technology, Chronology, and Significance*. Archaeopress, Oxford.

Sharon, G., 2009. Acheulian Giant-Core Technology: A Worldwide Perspective. *Current Anthropology* 50, 335–367.

Sharon, G., Feibel, C.S., Alperson-Afil, N., Harlavan, Y., Feraud, G., Ashkenazi, S., Rabinovich, R., 2010. New Evidence for the Northern Dead Sea Rift Acheulian. *PaleoAnthropology*, 79–99.

Shea, J.J., 2013. *Stone Tools in the Paleolithic and Neolithic Near East: A Guide.* Cambridge University Press, Cambridge.

Shimelmitz, R., 2015. The recycling of flint throughout the Lower and Middle Paleolithic sequence of Tabun Cave, Israel. *Quaternary International* 361, 34–45.

Shimelmitz, R., Kuhn, S.L., Jelinek, A.J., Ronen, A., Clark, A.E., Weinstein-Evron, M., 2014b. 'Fire at will': The emergence of habitual fire use 350,000 years ago. *Journal of Human Evolution* 77, 196–203.

Shimelmitz, R., Kuhn, S.L., Ronen, A., Weinstein-Evron, M., 2014a. Predetermined Flake Production at the Lower/Middle Paleolithic Boundary: Yabrudian Scraper-Blank Technology. *PLoS ONE* 9, e106293.

Shimelmitz, R., Kuhn, S.L., Weinstein-Evron, M., 2020. The evolution of raw material procurement strategies: A view from the deep sequence of Tabun Cave, Israel. *Journal of Human Evolution* 143, 102787.

Shipton, C., 2011. Taphonomy and Behaviour at the Acheulean Site of Kariandusi, Kenya. *African Archaeological Review* 28, 141–155.

Shipton, C., 2018. Biface Knapping Skill in the East African Acheulean: Progressive Trends and Random Walks. *African Archaeological Review* 35, 107–131.

Singer, B.S., 2014. A Quaternary geomagnetic instability time scale. *Quaternary Geochronology* 21, 29–52.

Smith, J.R., Giegengack, R., Schwarcz, H.P., 2004. Constraints on Pleistocene pluvial climates through stable-isotope analysis of fossil-spring tufas and associated gastropods, Kharga Oasis, Egypt. *Palaeogeography, Palaeoclimatology, Palaeoecology* 206, 157–175.

Smith, R.H., Dewey, H., 1914. The High Terrace of the Thames: Report on excavations made on behalf of the British Museum and H.M. Geological Survey in 1913. *Archaeologia* 65, 187–212.

Stanistreet, I.G., Stollhofen, H., Deino, A.L., McHenry, L.J., Toth, N.P., Schick, K.A., Njau, J.K., 2020. New Olduvai Basin stratigraphy and stratigraphic concepts revealed by OGCP cores into the Palaeolake Olduvai depocentre, Tanzania. *Palaeogeography, Palaeoclimatology, Palaeoecology* 554, 109751.

Stuart, A.J., 1992. The High Lodge Mamallian Fauna, in: Ashton, N.M., Cook, J., Lewis, S.G., Rose, J. (Eds.), *High Lodge: Excavations by G. de G. Sieveking, 1962-8 and J. Cook, 1988*. British Museum Press, London, pp. 120–123.

Tamrat, E., Thouveny, N., Taïeb, M., Opdyke, N.D., 1995. Revised magnetostratigraphy of the Plio-Pleistocene sedimentary sequence of the Olduvai Formation (Tanzania). *Palaeogeography, Palaeoclimatology, Palaeoecology* 114, 273–283.

Tauxe, L., Deino, A.D., Behrensmeyer, A.K., Potts, R., 1992. Pinning down the Brunhes/Matuyama and upper Jaramillo boundaries: a reconciliation of orbital and isotopic time scales. *Earth and Planetary Science Letters* 109, 561–572.

Taylor, D., Marchant, R., Hamilton, A.C., 2001. A reanalysis and interpretation of palynological data from the Kalambo Falls prehistoric site, in: *Kalambo Falls Prehistoric Site: Volume III. The Earlier Cultures: Middle and Earlier Stone Age*. Cambridge University Press, London, pp. 66–81.

Tester, P.J., 1951. Palaeolithic flint implements from the Bowman's Lodge Gravel Pit, Dartford Heath. *Archaeologia Cantiana* 63, 122–134.

Tester, P.J., 1965. An Acheulian site at Cuxton. *Archaeologia Cantiana* 63, 122–134.

Tester, P.J., 1975. Further consideration of the Bowman's Lodge industry. *Archaeologia Cantiana* 91, 29–39.

Toms, P., Hosfield, R.T., Chambers, J.C., Green, C.P., Marshall, P., 2005. Optical dating of the Broom Palaeolithic sites, Devon & Dorset (Centre for Archaeology Report No. 16/2005). English Heritage, London.

Trauth, M.H., Maslin, M.A., Deino, A., Strecker, M.R., 2005. Late Cenozoic Moisture History of East Africa. *Science* 309, 2051–2053.

Tryon, C.A., McBrearty, S., 2002. Tephrostratigraphy and the Acheulian to Middle Stone Age transition in the Kapthurin Formation, Kenya. *Journal of Human Evolution* 42, 211–235.

Tryon, C.A., McBrearty, S., Texier, P.-J., 2005. Levallois Lithic Technology from the Kapthurin Formation, Kenya: Acheulian Origin and Middle Stone Age Diversity. *African Archaeological Review* 22, 199–229.

Uribelarrea, D., Martín-Perea, D., Díez-Martín, F., Sánchez-Yustos, P., Domínguez-Rodrigo, M., Baquedano, E., Mabulla, A., 2017. A reconstruction of the paleolandscape during the earliest Acheulian of FLK West: The co-existence of Oldowan and Acheulian industries during lowermost Bed II (Olduvai Gorge, Tanzania). *Palaeogeography, Palaeoclimatology, Palaeoecology* 488, 50–58.

Voinchet, P., Despriée, J., Tissoux, H., Falguères, C., Bahain, J.-J., Gageonnet, R., Dépont, J., Dolo, J.-M., 2010. ESR chronology of alluvial deposits and first human settlements of the Middle Loire Basin (Region Centre, France). *Quaternary Geochronology* 5, 381–384.

Voinchet, P., Moreno, D., Bahain, J.-J., Tissoux, H., Tombret, O., Falguères, C., Moncel, M.-H., Schreve, D., Candy, I., Antoine, P., Ashton, N., Beamish, M., Cliquet, D., Despriée, J., Lewis, S., Limondin-Lozouet, N., Locht, J.-L., Parfitt, S., Pope, M., 2015. New chronological data (ESR and ESR/U-series) for the earliest Acheulian sites of north-western Europe. *Journal of Quaternary Science* 30, 610–622.

Walter, R.C., Manega, P.C., Hay, R.L., 1992. Tephrochronology of Bed I, Olduvai Gorge: An application of laser-fusion dating to calibrating biological and climatic change. *Quaternary International* 13–14, 37–46.

Washbourn, C.K., 1967. Lake levels and Quaternary climates in the eastern Rift Valley of Kenya. *Nature* 216, 672–673.

Wenban-Smith, F.F., 2004. Handaxe Typology and Lower Palaeolithic Cultural Development: Ficrons, Cleavers and Two Giant Handaxes from Cuxton. *Lithics* 25, 11–21.

Wenban-Smith, F.F., 2006. Cuxton giant handaxes. *Kent Archaeological Society Newsletter*, 68, pp. 2-3.

Wenban-Smith, F.F., 2013. *The Ebbsfleet Elephant: Excavations at Southfleet Road, Swanscombe in Advance of High Speed 1, 2003-4, Oxford Archaeology Monograph*. Oxford Archaeology, Oxford.

Wenban-Smith, F.F., Bates, M.R., Marshall, G., 2007. *Medway Valley Palaeolithic Project Final Report: The Palaeolithic Resource in the Medway Gravels (Kent)*. English Heritage, London.

Wenban-Smith, F.F., Bridgland, D.R., 2001. Palaeolithic archaeology at the Swan Valley Community School, Swanscombe, Kent. Proceedings of the Prehistoric Society 67, 219–225.

West, R.G., Gibbard, P.L., Boreham, S., Rolfe, C., 2014. Geology and geomorphology of the Palaeolithic site at High Lodge, Mildenhall, Suffolk, England. *Proceedings of the Yorkshire Geological Society* 60, 99–121.

Westaway, R., 2009. Quaternary vertical crustal motion and drainage evolution in East Anglia and adjoining parts of southern England: chronology of the Ingham River terrace deposits. *Boreas* 38, 261–284.

White, M.J., 2015. 'Dancing to the rhythms of the biotidal zone': settlement history and culture history in Middle Pleistocene Britain., in: Coward, F., Hosfield, R., Pope, M., Wenban-Smith, F. (Eds.), *Settlement, Society and Cognition in Human Evolution: Landscapes in the Mind*. Cambridge University Press, Cambridge, pp. 154–173.

White, M., Ashton, N., Bridgland, D., 2019. Twisted Handaxes in Middle Pleistocene Britain and their Implications for Regional-scale Cultural Variation and the Deep History of Acheulean Hominin Groups. *Proceedings of the Prehistoric Society* 85, 61–81.

White, T.S., Preece, R.C., Whittaker, J.E., 2013. Molluscan and ostracod successions from Dierden's Pit, Swanscombe: insights into the fluvial history, sea-level record and human occupation of the Hoxnian Thames. *Quaternary Science Reviews* 70, 73–90.
