# Supplementary material for: When is a handaxe a planned-axe? exploring morphological variability in the Acheulean
Source: PLoS One. 2024 Jul 16;19(7):e0307081. doi: 10.1371/journal.pone.0307081 (PMC11251633; doi:10.1371/journal.pone.0307081)
Supplement: Attachment — Submitted filename: Review Clark.pdf [file pone.0307081.s005.pdf]

“ When is a handaxe a planned-axe? Exploring morphological variability in the Acheulean", by James Clark, Ceri Shipton, Marie-Hélène Moncel, Philip R. Nigst, Robert A. Foley

The paper submitted by J. Clark et al. propose a comparison, based on a morphometric measurement, of more than 1.000 Lower Palaeolithic handaxes of Africa, Levant, and Europe. The authors choose a very specific method, that to be perfectly honest I'm not very familiar with. They have also chosen to cite only a very selective bibliographical reference, overwhelmingly from a specifical epistemological tradition. For example, there is almost no reference to lithic technology or use wear analysis. And they have every right to do so! But this makes the review rather complicated, especially the method section. Having said that, I will try to do a constructive review by questioning the compliance of the paper with scientific standards.

1. Introduction

The introduction is quite efficient, even if the definition of Acheulean (Acheulean = handaxes) is a bit of a caricature and could have been a little more nuanced (An extremely hot topic of debate!).

Then, the authors set out the various hypotheses that could explain the differences between handaxes but leave out the most obvious possibility: that they were different tools.

The research questions are exposed in the last paragraph “*This may better allow us to understand the reasons for handaxe persistence over time, and therefore how they related to the survival strategies of the hominins that produced and used them*.” This is in my opinion the biggest problem with the paper: how can this approach answer this question? How can the comparison between the shapes of the handaxes explain why they have lasted so long? Even though I'm repeating it, I'm not familiar with the method, this point needs to be clarified. How can the comparison of handaxes shapes contribute to the Acheulean issue?

2. Materials and Methods

The presentation of the archaeological sample is clear, well exposed in the Table 1. However, the question of the choices made is never addressed. Why these pieces and not others? Both chronologically and geographically.

Concerning the measurement strategy, I don't really have any comments, except that I'm wondering why the sections aren't considered, even though sections are crucial in the handaxe structure.

3. Results

The presentation of the raw results is clear and didactic. Unfortunately, when they try to interpret the results, things get complicated. The authors try to make correlations between morphometric data and thinning, refinement and resharpening which seems rather unexpected, and moreover on the basis of a few pieces from completely different contexts.

4. Discussion

The discussion is highly developed and puts the results into perspective. However, we move on from extremely general and partial results (some measurements on some piece) to micro-local considerations, difficult to reach in the Lower Palaeolithic context (environment, site function, subsistence activity, mobility, etc). This radical gap between this tzo scales seems difficult to reconcile and with contrary to the initial ambition of the paper.

5. Conclusion

Unfortunately, the results are disappointing: « *Instead, it seems likely that regional patterning emerges largely as the result of local norms of handaxe manufacture, and that these were, on average, more similar between neighbouring groups than more distant ones* ». Moreover, this result was expected given the lack of chronological and geographical diversity within each region.

To sum up, I would say that the work carried out is important and that it would be a shame not to publish the results. To be published, we suggest to

- discuss the relevance of the selected pieces: why we are comparing this handaxe with this other one?

- adapted the research question to the method: what results can we expect when we compare tool shapes?
