# Supplementary material for: When is a handaxe a planned-axe? exploring morphological variability in the Acheulean
Source: PLoS One. 2024 Jul 16;19(7):e0307081. doi: 10.1371/journal.pone.0307081 (PMC11251633; doi:10.1371/journal.pone.0307081)
Supplement: Attachment — Submitted filename: Comments_PlosOne.docx [file pone.0307081.s004.docx]

When is a handaxe a planned-axe? Exploring morphological variability in the Acheulean

I think this paper is really interesting and should be considered to be published within this journal. The topic is very attractive, as well as the sites included for the analysis. The data support the conclusions, the materials are appropriated, as well as the methods used. Statistically is well supported and the manuscript is well-written in a correct English. I think all the tables and figures and also adequate for the complete understanding of the paper, and even the Supplementary Materials. By all these reasons, I strongly recommend its publication with major revisions.

Materials and Methods

2.1 Sample: Authors said they have collected data from plan a d profile view, but then they only use plan-shape data to make the geometric morphometrics study. Profile views are only used from a metrical point of view. It is something key within this paper. Without any doubt, paper will be better justified and clearly written if profile-shape was explored.

Table 1. Other significant problem is no to include European sites from MIS11 where authors could test the coarse-grained materials: e.g. Menez Dregan, Terra Amata or other Spanish sites.

There is not technological analysis at all, explained in the Methods section. From my point of view, to make an accurate morphometrical analysis to have characterized technologically the sample. You should quantify the technological data to describe the materials. At least, it is necessary to include those aspects considered within this paper. What is resharpening? How has it been measured? What is thinning? How has it been measured?

There are no images, at all. You cannot check what the authors are explaining and this fact, combined with no technological analysis, made almost impossible to figure out what authors are referring to.

In this sense, it would be very helpful if Figure 3 could be integrated in the Figure 4, to visualize the morphological tendencies of each PCs.

Results

It is said that PC1 *“represents elongation and tip shape. Higher scores reflect more elongated and pointed handaxes, and lower scores less elongated and more ovate forms”.* Nevertheless, this PC represents also the variation in the whole width: distal width, mid width and proximal width. The Excel contains only one “width”, I assume the maximum width. It could be interesting to include the other widths. If you do this, you can calculate features such as the refinement of the tip (B1/T1), and reinforce or qualify your conclusions.

Here is where I would need to see the profile-shape analysis. I recommend strongly to introduce it. Using 2D images it is mandatory to make plan-shape and profile-shape analysis independently. But I am sure this paper will benefit a lot if authors accept to include this analysis.

3.1. Spatial variation. This title is quite intriguing. I suggest authors to change it by “variation in shape”/”shape variation” or something like this.

3.1.1. Shape = Plan-shape. It should be specified.

In the first line (265) symmetry is plan-symmetry. It should be specified along the whole test. Symmetry is traditionally associated to profile. As this is not the case, please clarify it.

Line 274-275: *Refinement can also be significantly distinguished between regions (F = 12.467, p = 4.969e-275 06), driven by relatively thicker handaxes in the African sample.* We cannot see it very well. I’d need the mean thickness by “area” and Supplementary Information has the Excel in a tool-by-tool list. It can also make reference to Figure 10. But if so, this Figure is reflecting major refinement in a great part of the African register (as recorded in Line 394). It seems to be contradictory.

Line 302 to 311. This is an interpretation of the data, and in a very personalized way. Perhaps all this paragraph should be moved to Discussion. But in any case, European sites are huge assemblages, too. Try to distinguish which sample is more palimpsest that the other is a mistake. Perhaps, it is better to consider that many African sites has a minimum age and most of the European sites have relative datings or big error ranges in absolute datings.

3.2.2 Symmetry = Plan-symmetry

3.2 Blank Type

It is necessary to include a Table with all information about the number of tools by type of tool, type of blank, etc.

Authors talk about cobble/flake. But what about the indeterminate blanks? A table would be so useful.

“Cobbles are overrepresented in Europe and Levant and are the thicker instruments”, so they introduce variability. But if you exclude cobbles to analyse flakes, it is something highly probable to find major uniformity, no? I think this subsection is a little bit redundant.

Fig 10. From Africa to Levant and Europe: those areas are chronologically complementary, mainly from MIS12, when all changes happens. In Europe there is an increase of diversity, major technological flexibility, also with the massive use of coarse-grained materials, together with a higher regionalization of technology. African register cannot show it because there are no included younger sites. On the contrary, if authors do not consider European sites with coarse-grained materials, you are avoiding a significant part of the register. As authors stated in lines 454-455, “*significant differences emerged driven by thicker artefact made on quartzite and lava”.*

Apart from Figure 11, it is necessary to see the distribution by zones but including cobble information.

3.4 Please, define Resharpening in the method section

3.4.2 Thinning: here is where a deep shape analysis of the profiles could be crucial. Because there is a clear association between thinning works and modification of the edge angle (García-Medrano et al. 2023).

Besides, from my point of view, the use of “cores” (choppers and discoidal cores) to compare with handaxes is not appropriated to get conclusions about resharpening and refinement. Basically, because resharpening is not the same than recurrence in flaking. This is the reason because the conclusions of this section are so obvious. Literally, authors said *“Unsurprisingly”.* (Line 567)

I think there is a mistake in the reference 42 (Line 565). It should be 44 or 45.

Regarding Figure 16, there are well-known classical sites in contradictory positions to what authors conclude form this Figure*: continuum of possible site-level reduction strategies, ranging from one more focused on the maintenance of tip shape and the production of flakes on the left side of the distribution, to one more focused on the production and maintenance of a sharp cutting edge on the right side of the distribution.*

Boxgrove or Elveden appear on the left and are the classical oval and tear-drop shape. On the contrary, warren Hill appears totally on the right, and it is well know that has both oval and pointed handaxes.

Besides the r value is not so high 0.302 to justify any strong correlation.

Please, consider to review in deep this site distribution.

Discussion

I think this section should carefully be reviewed by authors. It is necessary to order the ideas to be discussed and use the data presented in the result section to justify the conclusions. It is complicated to follow the structure and there are a lot of crucial aspects lost mainly in subsection 4.1.

Finding 5 in Table 3. According to Figure 10, there is a decrease in the African refinement, contrary to what it is stated here. The same in Line 687. Please review carefully this.
